# Acute effects of exercise snacks on postprandial glucose and insulin metabolism in adults with obesity: a systematic review and meta-analysis

**DOI:** 10.3389/fnut.2025.1708301

**Published:** 2025-11-20

**Authors:** Yuanbo Chang, Hai Wang, Xinbi Zhang, Haiyuan Liu

**Affiliations:** Capital University of Physical Education and Sports, Beijing, China

**Keywords:** sedentary behavior, exercise snacks, interrupting sitting, obesity, glucose iAUC, insulin

## Abstract

**Objective:**

To quantify the acute effects of brief, frequent interruptions to prolonged sitting (“exercise snacks”) on postprandial glucose and insulin in adults with obesity, and to explore potential effect modifiers.

**Data sources and methods:**

Following PRISMA 2020, seven sources (PubMed, Web of Science, Cochrane Library, Embase, ClinicalTrials.gov, ICTRP, CINAHL) were searched to July 10, 2025. Randomized crossover or parallel trials in adults with obesity comparing activity breaks with uninterrupted sitting were included. Co-primary outcomes were glucose and insulin incremental area under the curve (iAUC); secondary outcomes were total AUC (tAUC) and mean levels. Random-effects meta-analyses synthesized standardized or mean differences (95% CI); heterogeneity was quantified by I^2^. Prespecified subgroup and sensitivity analyses were undertaken; small-study effects were examined when k ≥ 10. Risk of bias was appraised with Cochrane RoB 2.0 (fixed-effect estimates were additionally inspected when heterogeneity was low to moderate).

**Results:**

Seventeen trials (261 unique participants; predominantly randomized crossover) were included. Versus uninterrupted sitting, activity breaks reduced glucose iAUC (SMD = −0.49, 95% CI –0.85 to −0.14; I^2^ = 76%) and reduced insulin iAUC (SMD = −0.26, 95% CI –0.50 to −0.03; I^2^ = 44%). Glucose tAUC and mean glucose showed non-significant downward trends. Mean insulin decreased (SMD = −0.54, 95% CI –0.97 to −0.10), albeit with high heterogeneity (I^2^ = 76%). Exploratory subgroup analyses suggested larger effects with higher-frequency (≤30-min) and short-bout (≤3-min) interruptions and with walking or simple resistance, although tests for subgroup differences were generally non-significant. Meta-regressions showed age predicted glucose iAUC, BMI and interruption frequency predicted mean insulin, no moderator predicted insulin iAUC, and intervention intensity (daily MET) had minimal, non-significant effects. Findings were robust in leave-one-out and model-assumption sensitivity analyses, with no clear small-study effects for glucose outcomes.

**Conclusion:**

In adults with obesity, interrupting sitting about every ≤30 min with 2–5 min of light-to-moderate walking or simple resistance acutely attenuates postprandial glucose and insulin responses. These findings support exercise “snacks” as a pragmatic behavioral strategy, while longer-term randomized trials are needed to define durability and refine dose parameters.

**Systematic review registration:**

https://www.crd.york.ac.uk/PROSPERO/view/CRD420251144139, Identifier CRD420251144139.

## 1 Introduction

Sedentary behavior, defined as any waking activity performed in a sitting or reclining posture with an energy expenditure of ≤1.5 metabolic equivalents (METs), has become one of the most prevalent lifestyle patterns in modern society (1–3). According to epidemiological surveys by the World Health Organization (WHO), more than one-quarter of adults and nearly 80% of adolescents worldwide fail to achieve the recommended levels of physical activity, and prolonged sedentary time is increasingly recognized as an independent health risk, beyond insufficient exercise (4–6). A large body of longitudinal evidence indicates that chronic sedentary behavior is closely associated with insulin resistance, impaired glucose tolerance, and increased incidence of type 2 diabetes and metabolic syndrome (7–10). Individuals with obesity are particularly vulnerable in this process, as obesity itself represents a high-risk state for insulin resistance and chronic low-grade inflammation, and is strongly linked to elevated risks of diabetes, cardiovascular disease, and other metabolic disorders (11–13). Consequently, identifying practical and feasible strategies to interrupt prolonged sitting and alleviate the metabolic burden in obese populations has become a pressing public health priority.

In recent years, the concept of brief “exercise snacks”—short, frequent bouts of light-to-moderate activity or standing embedded within prolonged sitting—has attracted growing attention (14). The physiological mechanisms underlying this intervention are primarily related to its ability to improve insulin sensitivity, enhance glucose metabolism, and increase fat oxidation. Short bouts of activity stimulate skeletal muscle contractions, which in turn increase glucose uptake and reduce insulin resistance (15). This effect is particularly important for individuals with obesity, who typically suffer from chronic low-grade inflammation and metabolic dysfunction (16). Additionally, brief physical activity breaks may activate the sympathetic nervous system, leading to improved vascular function and reduced postprandial glucose spikes (17). These physiological responses form the basis for the observed improvements in cardiometabolic health, particularly when sitting is interrupted by short, frequent activity breaks. Accumulating randomized crossover trials indicate that interrupting sitting with such activity/standing breaks improves cardiometabolic control (18, 19), with meta-analytic evidence showing moderate acute reductions in postprandial glucose and insulin versus uninterrupted sitting (e.g., pooled SMD for glucose −0.54 and for insulin −0.56) (20). These effects have been demonstrated across modalities (e.g., walking, standing, simple resistance) and in both laboratory and free-living contexts, supporting the translational potential of sedentary/activity breaks (21). Importantly, dose features appear to matter: a recent three-level meta-analysis comparing interruption schedules reported that higher-frequency breaks (≤30 min per bout) achieved greater acute glucose lowering than lower-frequency protocols (>30 min per bout), whereas differences for insulin, triglycerides, blood pressure, and vascular function were not statistically significant and the certainty of evidence was low (21). Current guidelines increasingly encourage “sit less, move more,” and some diabetes guidance suggests interrupting sitting every 30 min, though this specific interval derives from limited crossover evidence rather than head-to-head frequency trials—underscoring the need for rigorous dose-optimization research (21).

However, despite these encouraging signals, the evidence base remains incomplete in ways directly relevant to clinical translation in adults with obesity. First, most trials have enrolled metabolically healthy or mixed-weight samples, and dedicated investigations in adults with obesity remain limited in number, size, and duration (22). Second, substantial heterogeneity in interruption protocols—including modality (e.g., walking, standing, simple resistance), frequency (≤30 vs. > 30 min), bout duration (≤3 vs. > 3 min), and total daily volume (≤30, 31–60, 61–120, >120 min/day)—impedes comparability and likely contributes to between-study inconsistency (23, 24). Third, it is unclear which subgroups (e.g., sex, age, BMI category) and which dose features (frequency, bout duration, daily volume) yield the largest improvements in postprandial glucose and insulin (25, 26). These gaps directly motivate the present systematic review and meta-analysis, which aims to examine the acute effects of exercise-snack interventions on postprandial glucose and insulin metabolism in adults with obesity. The study uses glucose and insulin iAUC as co-primary outcomes. Beyond quantifying overall effects, subgroup analyses were conducted to explore how intervention characteristics (type, frequency, duration, intensity, and total dose) and participant characteristics (sex, age, degree of obesity) modulate these effects. The goal is to identify the most effective strategies and provide an evidence base for individualized lifestyle interventions and future public health recommendations.

## 2 Methods

This systematic review followed the PRISMA 2020 statement (27), and was registered in PROSPERO (CRD420251144139).

### 2.1 Search strategy

We systematically searched PubMed, Web of Science (Core Collection), Cochrane Library, Embase, ICTRP, ClinicalTrials.gov, and CINAHL from inception to July 10, 2025. Only peer-reviewed articles published in English were considered, with no restrictions on publication date. To maximize sensitivity, we applied a comprehensive search strategy combining MeSH terms and free-text keywords (e.g., Cochrane Library strategy shown in Supplementary Table S1). In addition to database searching, we conducted three supplementary steps: (1) manual screening of reference lists of eligible studies, (2) citation tracking of included articles, and (3) reviewing relevant systematic reviews to identify additional studies. We also searched PROSPERO and the Cochrane Database of Systematic Reviews to ensure no similar reviews had already been published.

### 2.2 Study selection

All retrieved records were first de-duplicated manually using Zotero (version 7.0). After de-duplication, two reviewers independently screened titles and abstracts according to the pre-specified inclusion and exclusion criteria. Any disagreements were resolved through discussion; if consensus could not be reached, a third independent reviewer was consulted to adjudicate. Finally, two reviewers independently assessed the full texts to confirm eligibility of the included studies.

### 2.3 Eligibility criteria

Eligibility criteria were prespecified according to the PICOS framework. We included randomized parallel-group or randomized crossover trials enrolling adults (≥18 years) with obesity. Obesity was operationalized *a priori* as (i) BMI ≥ 30 kg/m^2^ for European/White populations or ≥27.5 kg/m^2^ for Asian populations (28, 29), or (ii) BMI 27.0–29.9 kg/m^2^ with phenotypic obesity, evidenced by central adiposity [waist circumference ≥102 cm in men or ≥88 cm in women (30), or ethnic-specific cut-offs] or excessive body fat [≥35% in women, ≥25% in men (31)]. When BMI was not reported, body-fat percentage or waist circumference meeting the above thresholds was accepted. Trials with mixed weight status were eligible only when ≥80% of participants met the obesity criteria or when data for participants with obesity were extractable. No upper age limit was applied.

Interventions were structured strategies to interrupt prolonged sitting (e.g., exercise snacks, brief activity breaks, accumulated light-to-moderate activity) delivered under laboratory or free-living conditions; comparators involved uninterrupted/usual sitting.

The primary outcomes were postprandial glucose and insulin incremental area under the curve (iAUC). Secondary outcomes included other indices of glucose metabolism, such as total AUC, mean glucose or insulin levels. We excluded studies involving participants with type 1 or type 2 diabetes, metabolic syndrome, cardiovascular disease, or other chronic illnesses; non-obese or mixed populations without separate data for obese individuals; interventions unrelated to sedentary interruption or without sufficient detail on dose parameters; outcomes unrelated to glucose metabolism or without quantitative data; and non-randomized, observational, review, abstract, or non–peer-reviewed publications.

### 2.4 Data extraction

Two reviewers independently extracted data using a standardized Excel form. Extracted information included: author, country, year of publication; participant characteristics (sample size, age, BMI, health status); intervention details (type, timing, frequency, duration, and total volume of activity); supervision status; outcome measures; and measurement devices. Discrepancies were resolved by a third independent reviewer. For studies with missing data, we first attempted to contact the authors. If no response was obtained, data were extracted from figures using WebPlotDigitizer 4.1, a tool validated for high reliability and accuracy (32). When studies reported results in formats other than mean and standard deviation (33) (e.g., confidence intervals or standard errors), values were converted to standard deviations using established statistical methods. Studies for which essential data could not be retrieved were excluded from the quantitative analysis.

### 2.5 Risk of bias assessment

Two independent reviewers assessed the methodological quality of the included studies using the Cochrane Collaboration’s Risk of Bias (RoB 2.0) tool (34). The assessment covered domains including random sequence generation, allocation concealment, blinding, completeness of outcome data, selective reporting, and other potential sources of bias. Each domain was rated as “low risk,” “high risk,” or “unclear risk.” Disagreements were resolved through discussion or by consulting a third reviewer.

### 2.6 Certainty of the evidence

The effectiveness evidence for each outcome was assessed using the GRADE framework, which rated the certainty as high, moderate, low, or very low (35). This rating was based on factors such as risk of bias, consistency, imprecision, and publication bias. GRADE evaluation was performed by two independent researchers, and discrepancies were resolved through consensus.

Evidence quality was assessed using the following criteria: (1) Risk of Bias: Evidence was downgraded by one level if there were some concerns about bias, and by two levels if the risk of bias was classified as high. (2) Inconsistency: The impact of statistical heterogeneity (I^2^) was considered. Evidence was downgraded by one level if the I^2^ value was moderate (> 25%), and by two levels if the I^2^ value was high (> 75%). (3) Imprecision: Evidence was downgraded by one level if the statistical power of the studies was less than 80% or if there was no clear direction of the effects (36). (4) Risk of Publication Bias: Evidence was downgraded by one level if Egger’s test indicated a *p*-value of less than 0.05, suggesting potential publication bias.

### 2.7 Data analysis

Due to the limited number of studies reporting glycemic variability outcomes (e.g., MAGE, SD, CV, TIR), these indicators were not included in the meta-analysis. Instead, the analysis focused on glucose outcomes (mean, incremental area under the curve [iAUC], and total area under the curve [tAUC]) and insulin outcomes (mean, iAUC, and tAUC). Given that most studies employed continuous glucose monitoring (CGM), which provides a more comprehensive assessment of postprandial glycemic fluctuations, we prioritized iAUC as the primary endpoint, as it is considered the most sensitive indicator of postprandial glycemic responses. When iAUC data were not available, tAUC or mean values were used as substitutes.

All continuous outcomes were pooled as mean differences (MDs) or standardized mean differences (SMDs) with 95% confidence intervals (CIs). Because of methodological heterogeneity across studies (e.g., differences in intervention type, frequency, bout duration, and total dose), random-effects models (REMs) were applied. Between-study heterogeneity was assessed using Cochran’s Q (χ^2^) test and the I^2^ statistic. Following a conservative criterion, we considered heterogeneity statistically significant at *p* < 0.05 on the Q test, and substantial when I^2^ ≥ 50% (37, 38).

Sensitivity analyses were conducted using the leave-one-out method. Publication bias was assessed for primary outcomes (glucose iAUC, glucose tAUC) using Begg’s and Egger’s tests. For outcomes with fewer than 10 studies, only funnel plots were generated for qualitative assessment.

### 2.8 Subgroup analysis

To further explore potential sources of heterogeneity and effect modifiers, we conducted pre-specified subgroup analyses. Subgroups were defined based on participant characteristics—sex (female, male, mixed), age (<30 years vs. ≥30 years), and BMI category (mild obesity [BMI < 32 kg/m^2^] vs. moderate-to-severe obesity [BMI ≥ 32 kg/m^2^])—as well as intervention characteristics, which were derived from the characteristics of the included studies. These intervention characteristics included activity type (standing, walking, resistance exercise, cycling, stair climbing, running, leg fidgeting), break frequency (≤30 min vs. >30 min), bout duration (≤3 min vs. >3 min), total daily dose of interruption (≤30 min/day, 31–60 min/day, 61–120 min/day, >120 min/day), and intervention intensity, as measured by the regression analysis conducted on the intervention characteristics. These subgroup analyses were designed to clarify “for whom” and “under what conditions” sedentary interruption strategies are most effective. Given the limited number of studies in some subgroups, these analyses should be considered exploratory.

## 3 Results

### 3.1 Search results

**Figure 1:**
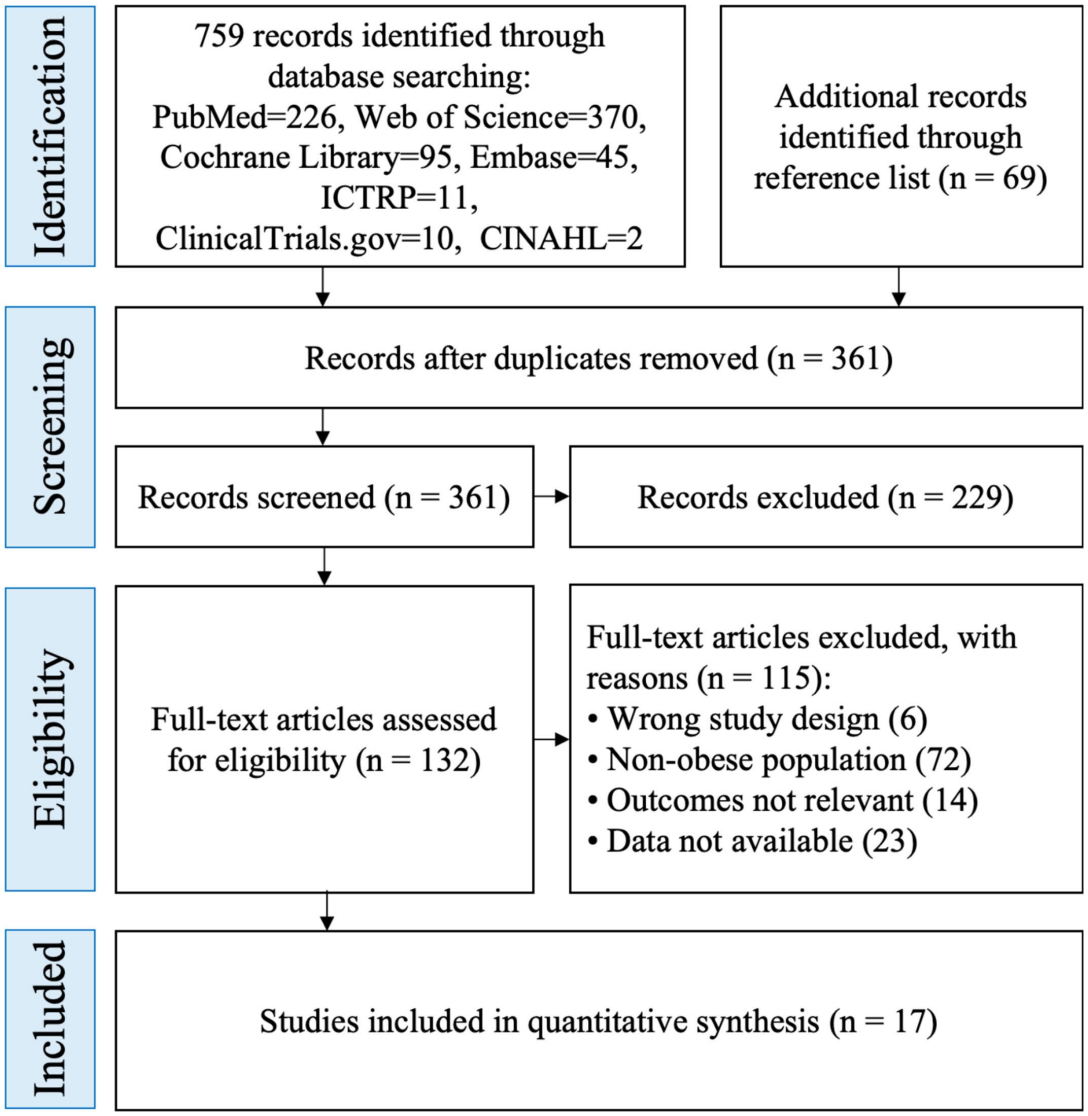
PRISMA flowchart.

We retrieved 759 records from five bibliographic databases and two trial registries (PubMed = 226, Web of Science = 370, Cochrane Library = 95, Embase = 45, CINAHL = 2, ICTRP = 11, ClinicalTrials.gov = 10) and identified 69 additional records through reference lists, yielding 828 records in total. After de-duplication (*n* = 467), 361 unique records remained. Title/abstract screening excluded 229 records, leaving 132 articles for full-text assessment. Of these, 115 were excluded (wrong study design = 6; non-obese population = 72; outcomes not relevant = 14; data not available = 23). Seventeen randomized controlled or crossover trials were included in the quantitative synthesis (Figure 1).

### 3.2 Characteristics of included studies

Seventeen randomized crossover trials comprising 261 unique participants were included, of whom 128 (49%) were women. Owing to the crossover design, individual participants contributed multiple outcome measures under different intervention conditions, leading to a higher number of observations than independent participants. Sample sizes ranged from 8 to 28, with mean ages between 21 and 59.6 years and BMI values ranging from 27.1 to 38. Notably, five studies enrolled participants whose BMI did not strictly meet the conventional obesity thresholds (≥30 kg/m^2^ for Europeans, ≥28 kg/m^2^ for Asians). Among these, one study classified obesity based on body fat percentage exceeding accepted cutoffs (39), while the other four included individuals at borderline BMI levels (23, 40–42). These borderline participants, although not meeting the strict BMI criteria for obesity, were included due to their proximity to the established diagnostic thresholds and the recognized metabolic risks associated with these BMI levels. This inclusion is scientifically justified, as individuals with BMI values near the diagnostic cutoffs often present similar health risks, including insulin resistance and metabolic dysfunction, which are relevant to the effects of exercise interventions. Therefore, their inclusion enhances the generalizability and applicability of the findings to a broader population at risk. Control conditions involved uninterrupted sitting for 3 to 10 h.

Intervention characteristics varied. The most frequently applied activity breaks were resistance exercises (34.2%) (41–45), walking (22.2%) (43, 46–50), and cycling (15.4%) (51), followed by standing (11.1%) (23, 40, 52), stair climbing (6.8%) (53), running (6.8%) (39), and leg fidgeting (3.4%) (54). Break frequency was most often every 30 min (64.2%), with fewer studies using 20 min (11.7%) (42, 46) or 60 min (9.2%) (40, 47, 53) intervals; rarer protocols included 2.5 min (4.2%) (54), 120 min (3.3%) (53), 180 min (3.3%) (39), 45 min (1.7%) (43), 3 min (1.7%) (23), and 510 min (0.8%) (43). Bout duration most commonly lasted 3 min (37.3%) (41–44, 47–49), 5 min (15.1%) (50, 51), or 2 min (11.9%) (39, 46, 50, 53). Shorter bouts included 30 s (4.8%) (45) or 1.5–2.5 min (7.1%) (23, 39, 46, 48, 50, 53, 54), while longer bouts extended to 10 min (3.2%) (39), 15 min (1.6%) (23), or 30 min (0.8%) (45). One study adopted a progressive protocol, interrupting sitting hourly with bouts increasing from 10 to 30 min, totaling 150 min/day (0.8%) (32). Detailed study characteristics are summarized in Table 1.

**Table 1:** Characteristics of included trials on interrupting prolonged sitting in adults with overweight or obesity.

Study	Participants	Protocol	Outcomes	Measurement	Key results
Hoffmann et al. (2024) (40) (Germany)	17 (8F/9M); Age 23.4 ± 3.3 y; BMI 29.7 ± 3.8	Acute 4-arm crossover; SIT: 9 h uninterrupted sitting; SIT-STAND: alternating sitting/standing, 8 bouts/day (10–30 min each, total 150 min/day); STAND: continuous standing (8 h); WALK: slow treadmill walking (1 mph, 8 h); meals standardized, full supervision.	8-h mean glucose; 2-h postprandial glucose (breakfast, lunch); HR, HRV (SDNN, RMSSD, LF/HF)	Capillary blood (earlobe) + 24-h Holter ECG	8-h mean glucose ↓ in SIT-STAND, STAND, WALK vs. SIT (WALK significant); 2-h post-breakfast glucose ↓ in WALK vs. SIT; no lunch effect; HRV improved in WALK, mixed in STAND.
Gao et al. (2024) (43) (China)	18 (0F/18M); Age 21.0 ± 1.2 y; BMI 28.8 ± 2.2	Randomized 4-arm crossover (8.5 h each). SIT: uninterrupted sitting; ONE: single 30-min treadmill walk at 4 km/h, 1 h after breakfast; WALK: 3-min treadmill walk every 45 min × 10 (total 30 min); SQUAT: 3-min squats every 45 min × 10 (total 30 min). All conditions had standardized meals (breakfast ≈600 kcal, lunch ≈1,100 kcal). EE matched across active conditions; washout ≥7 days.	Primary: 8.5-h glucose net incremental AUC (netiAUC). Secondary: EMG (aEMG, activity duration) of quadriceps, hamstrings, gluteals.	CGM (Abbott FreeStyle Libre); EMG shorts (quadriceps, hamstrings, gluteals).	Glucose netiAUC ↓ in ONE, WALK, SQUAT vs. SIT (all *p* < 0.05). Greater ↓ in WALK & SQUAT vs. ONE (*p* < 0.05). Quadriceps aEMG ↑ in WALK; gluteal aEMG ↑ in SQUAT.
Gale et al. (2024) (41) (New Zealand)	28 (20F/8M); Age 25.5 ± 5.6 y; BMI 29.2 ± 6.9	Randomized 2-arm crossover; SIT: 4 h uninterrupted evening sitting (~17:30–21:30); RAB: SIT interrupted with 3-min simple resistance exercise (chair squats, calf raises, standing knee raises with hip extensions) every 30 min × 8; standardized dinner (34% daily energy) + dessert (10% energy); washout ≥6 days.	Mean interstitial glucose; total AUC; positive iAUC; glycemic variability (CONGA-1, SD glucose).	CGM (Freestyle Libre Pro, Abbott).	During 4-h intervention: RAB ↓ mean glucose (−8.3%), AUC (−8.9%), iAUC (−33%) vs. SIT (all *p* < 0.01). No sustained effects overnight or at 24–48 h. RAB ↑ glycemic variability indices (CONGA-1, SD glucose) during nocturnal and 24–48 h periods.
Gale et al. (2023) (44) (UK)	10 (7F/3M); Age 26.8 ± 5.8 y; BMI 36.6 ± 5.5	Randomized 2-arm crossover; SIT: 4 h uninterrupted evening sitting (~17:00–21:00); RAB: 3-min simple resistance exercises (chair squats, calf raises, standing knee raises with hip extensions) every 30 min × 8 (24 min total). Standardized dinner (34% daily energy) + dessert (10% energy). Washout ≥6 days.	Plasma glucose, insulin, triglycerides (AUC, iAUC over 4 h)	Venous blood samples (hourly + 30/45 min post meals); assays via Roche Diagnostics	In obese group: RAB ↓ glucose iAUC (−20.6% vs. SIT); insulin iAUC ↓14.5% (NS); triglyceride AUC + 6.1% (NS).
Bailey et al. (2022) (52) (UK)	12 (8F/4M); Age 48 ± 10 y; BMI 33.3 ± 5.5	Randomized crossover; two 4-day regimens under free-living conditions. SIT: ≥10 h sitting/day, including ≥7 bouts ≥1 h; ≤1.5 h standing/stepping per day. INTERRUPTED: break sitting ≥every 30 min during ≥10 waking h with 3–5 min activity (standing, walking, simple resistance, stairs, sit-to-stand); accumulate 6–10 min activity each hour; ≥1.5 h/day standing/PA. Diet standardized (replicated intake across regimens).	24-h mean glucose; total AUC; net iAUC; glucose variability (CV).	CGM (FreeStyle Libre, Abbott); activity measured via activPAL3 (sitting/standing/stepping).	No significant differences between regimens: 24-h mean glucose, AUC, iAUC, and CV ↔. Sitting time ↓58 min/day and prolonged bouts (≥30, ≥60 min) ↓99 and 63 min/day, stepping ↑40 min/day in INTERRUPTED regimen, but no glucose improvements.
Wongpipit et al. (2021) (47) (Hong Kong, China)	21 (0F/21M); Age 23 ± 4 y; BMI 29.8 ± 3.2; WC 98.7 ± 7.1 cm	Randomized 3-arm crossover (7-h trials, ≥7-d washout). SIT: uninterrupted sitting; 3-min: light walking (3.2 km/h) for 3 min every 30 min (10 bouts, total 30 min); 6-min: light walking (3.2 km/h) for 6 min every 60 min (5 bouts, total 30 min). Standardized mixed meals at 0 h and 3 h.	Primary: 6-h glucose tAUC, iAUC; insulin tAUC, iAUC. Secondary: triglyceride tAUC, iAUC; NEFA tAUC, iAUC.	Venous blood sampling (baseline and every 30–60 min up to 6 h); glucose (Biosen-C), insulin (Mercodia ELISA), triglycerides & NEFA (Randox colorimetric kits).	Glucose & insulin tAUC/iAUC ↔ across conditions. Triglyceride tAUC ↓3.7% (3-min) and ↓11% (6-min) vs. SIT; iAUC ↓13% (3-min) and ↓20% (6-min) (all *p* < 0.05). NEFA ↔.
Wanders et al. (2021) (48) (Netherlands)	24 (19F/5M); Age 60 ± 8 y; BMI 30.2 ± 2.5	Randomized 4-arm crossover (≥1-wk washout). SIT: 4 h uninterrupted sitting; ACT: sitting interrupted with 5-min cycling every 30 min (total 30 min, 50–70% HRmax). Both SIT and ACT combined with one of two breakfasts: HPLF (high-protein/low-fat, 438 kcal, 11% fat, 31% protein, 52% carb, incl. Wholemeal bread + blueberries) vs. WEST (Western-style, 439 kcal, 39% fat, 14% protein, 45% carb, incl. White bread + jam).	Cognitive performance (TAP: alertness, flexibility, working memory); Perceivable benefits (mood, sleepiness, hunger); Vascular (carotid artery reactivity, BP); Metabolic (glucose, insulin, lipids).	Venous blood (hourly for 4 h); cognitive tests (TAP battery); mood (POMS); vascular ultrasound + BP monitor.	PA breaks ↓ postprandial insulin iAUC vs. SIT (*p* = 0.004), independent of meal type. Glucose iAUC ↑ after WEST vs. HPLF (*p* = 0.01), unaffected by PA breaks. PA breaks improved mood (↓ TMD, fatigue, sleepiness; ↑ vigor) but ↔ cognitive and vascular outcomes. Lipids (TG, cholesterol, HDL, LDL) ↔.
Smith et al. (2021) (49) (Sweden)	16 (10F/6M); Age 50 [44–53] y; BMI 32 [32–35.8]	Parallel-group RCT (4 wk). Baseline: 1-wk habitual living. Control: maintain habitual lifestyle. FABS: smartwatch prompts every 30 min (08:00–18:00) to perform 3-min low-to-moderate PA (walking, stair-climbing, squats; ≥15 steps counted as a break). Participants asked to keep diet stable; free-living setting.	OGTT: glucose, insulin (iAUC, HOMA2-IR, Matsuda, HIRI); Fasting glucose, insulin, HbA1c, lipids; 24-h interstitial glucose (mean, SD, CV, CONGA); Skeletal muscle lipidomics.	CGM (FreeStyle Libre); activPAL for activity; venous blood (clinical chemistry, OGTT); skeletal muscle biopsies (vastus lateralis, lipidomics).	FABS ↓ fasting glucose (−0.34 ± 0.37 mmol/L, *p* = 0.037) and ↓ glucose variability (%CV –2%, *p* = 0.039) vs. baseline; glucose tolerance (OGTT AUC) ↔; insulin sensitivity indices ↔; LDLc trend ↓ (*p* = 0.078); skeletal muscle lipidome largely unchanged (2 TG ↑, overall profile stable).
Pettit-Mee et al. (2021) (54) (USA)	20 (15F/5M); Age 42 ± 3 y; BMI 37.5 ± 2.1	Randomized 2-arm crossover (≥7-d washout). After 75 g oral glucose, participants sat for 3 h under two conditions: No-fidget (uninterrupted sitting) vs. Leg-fidget (alternate 2.5 min rest / 2.5 min bilateral leg fidgeting throughout 3 h).	3-h glucose tAUC, iAUC; insulin tAUC, iAUC; Matsuda ISI; accelerometer counts; VO₂; popliteal artery blood flow.	Venous blood: glucose (YSI 2300), insulin (ALPCO ELISA); indirect calorimetry (TrueOne 2,400); Doppler ultrasound (popliteal artery); accelerometer (ActiGraph GTX3).	Fidgeting ↓ glucose tAUC, iAUC (*p* < 0.05); ↓ insulin tAUC (*p* < 0.05), insulin iAUC ↔; Matsuda ISI ↑; accelerometer counts, VO₂, and popliteal artery blood flow ↑.
Hawari et al. (2019) (45) (UK)	14 (11 M/3F); Age 37 ± 16 y; BMI 30.5 ± 3.8; WC 102.3 ± 10.7 cm	Randomized 2-arm crossover (6.5 h). SIT: uninterrupted sitting. SIT/STAND: every 20 min perform 10 chair squats (~30 s, sit-to-stand transitions). Standardized breakfast (8 kcal/kg; 37% fat, 49% CHO, 14% protein) at baseline and identical lunch at 3.5 h.	Energy expenditure; substrate utilization (CHO, fat oxidation); plasma glucose, insulin, triglycerides.	Indirect calorimetry (Douglas bags); venous blood (YSI 2300 for glucose, Mercodia ELISA for insulin, Randox enzymatic kit for TG).	SIT/STAND ↑ total EE (+410 kJ, +16.6%) and CHO oxidation; fat oxidation ↑ post-breakfast only. Post-breakfast insulin ↓10.9% vs. SIT (*p* = 0.047). Glucose and TG ↔.
Rodriguez-Hernandez et al. (2018) (50) (USA)	10 (10F/0M); Age 36 ± 5 y; BMI 38.0 ± 1.6; Body fat 49.6 ± 1.4%	Randomized 3-arm crossover (4 h; ≥48 h washout). SED: uninterrupted sitting. SED+2 min: 2-min moderate-intensity walking every 30 min (16 min total). SED+5 min: 5-min moderate-intensity walking every 30 min (40 min total). Standardized breakfast cereal + rice milk at baseline.	Interstitial glucose: 2-h and 4-h postprandial glucose AUC, iAUC.	CGM (iPro2, Medtronic; readings every 5 min, calibrated by capillary glucose).	2-h postprandial glucose iAUC ↓ in SED + 5 min vs. SED (*p* = 0.005, d = −0.57). 2-min walking ↓ iAUC (NS, *p* = 0.086). 4-h glucose AUC ↔ across conditions.
Gay et al. (2018) (53) (USA)	9 (7F/2M); Age 54 ± 7 y; BMI 30.0 ± 3.1; Body fat 38.1 ± 7.7%;	Randomized 3-arm crossover (8 h, consecutive days). CON: sedentary control. 2-min: stair climbing (vigorous, 60–85% HRR) 2 min every hour × 8 (16 min total). 4-min: stair climbing 4 min every 2 h × 4 (16 min total). Standardized meals (65% CHO, 25% fat, 10% protein).	CGM-derived interstitial glucose (5-min intervals); 12-h and 2-h post-meal AUC; post-exercise change in glucose (0–30 min).	CGM (iPro2, Medtronic), calibrated by capillary glucose (OneTouch UltraMini); accelerometer (ActiGraph GT3X+).	12-h glucose AUC ↔ across conditions. 4-min stair climbing ↓ glucose at 30 min post-exercise vs. CON (Cohen’s d = −0.91). No effect for 2-min bouts (d = −0.13). Effects depended on pre-exercise glucose: ↓ only when ≥90 mg/dL; ↔ when <90 mg/dL.
Climie et al. (2018) (42) (Australia)	9 (5 M/4F); Age 32 ± 3 y; BMI 29.7 ± 4.1; WC 94 ± 10 cm	Randomized 2-arm crossover (3.5 h). SIT: uninterrupted sitting while watching TV. ACB: every 20 min perform 3 min of light-intensity body-weight activities (half-squats, calf raises, knee raises, glute contractions). Standardized dinner (≈45% daily energy; 53–55% CHO, 12–15% protein, 30–33% fat).	Plasma glucose & insulin (iAUC); interstitial glucose (tAUC); glycemic variability (CONGA-1, SD, MAGE); triglycerides; BP; perceived fatigue & hunger.	Venous blood (glucose: hexokinase; insulin: RIA; TG: Abbott enzymatic); CGM (Medtronic iPro2, 5-min intervals, calibrated by capillary glucose); BP monitor (Omron).	ACB ↓ plasma glucose iAUC (−33%, *p* = 0.019) and insulin iAUC (−41%, *p* = 0.033) vs. SIT. Interstitial glucose tAUC ↓ during ACB (*p* < 0.001), but not pre-sleep/nocturnal. Glycemic variability (CONGA-1 ↓, *p* = 0.002); SD and MAGE ↓ during condition but lost significance after adjustment. TG and BP ↔. Fatigue ↓ in ACB.
Hawari et al. (2016) (23) (UK)	10 (10 M/0F); Age 33 ± 13 y; BMI 28.3 ± 3.0; WC 100.2 ± 9.5 cm	Randomized 3-arm crossover (8 h). SIT: uninterrupted sitting. PRO-Stand: stand 15 min every 30 min (total 4 h standing). INT-Stand: 10 × 90 s standing + 30 s sitting cycles per 30 min (total 4 h standing, 160 sit-to-stand transitions). Standardized breakfast (8 kcal/kg; 37% fat, 49% CHO, 14% protein) and identical lunch after 4 h.	Energy expenditure; substrate utilization (CHO, fat oxidation); plasma glucose, insulin, triglycerides (AUC).	Indirect calorimetry (Douglas bags); venous blood (YSI 2300 glucose, Mercodia ELISA insulin, Randox enzymatic TG).	INT-Stand ↑ EE (+20.4%) vs. SIT and PRO-Stand (*p* < 0.001); PRO-Stand ↑ EE (+10.7%) vs. SIT (*p* < 0.001). INT-Stand ↑ fat oxidation vs. SIT (+20.2%, *p* < 0.01); CHO oxidation ↑ in both PRO-Stand and INT-Stand. Glucose, insulin, TG responses ↔ across conditions.
Larsen et al. (2015) (46) (Australia)	19 (11 M/8F); Age 56.7 ± 1.5 y; BMI 32.7 ± 1.0; WC 108.3 ± 2.7 cm	Randomized 2-arm crossover (3 days, outpatient). SIT: 7-h/day uninterrupted sitting ×3 days. BREAKS: 2-min light walking (3.2 km/h) every 20 min (17 bouts/day; total ~34 min) × 3 days. Controlled standardized diet across conditions. MTT (75 g CHO, 50 g fat) on day 1 and day 3.	Plasma glucose, insulin, triglycerides (tAUC, iAUC); insulin sensitivity indices (HOMA-IR, HOMA-*β*%, MISI).	Venous blood (hourly during 4-h MTT; Abbott Architect ci16200 for glucose, insulin, TG).	BREAKS ↓ glucose iAUC by ~31–32% vs. SIT (days 1 & 3, *p* = 0.001). Insulin iAUC ↓ ~ 15% vs. SIT (*p* = 0.01); insulin tAUC ↓12% (*p* = 0.009). Triglycerides ↔; HOMA-IR & MISI ↔; HOMA-β% ↑ over time (condition-independent).

Data are summarized for each trial, including study population, intervention protocol, primary outcomes, measurement methods, and key findings (direction of effects). Participants are reported as *N* (female/male), Age (mean ± SD or median [IQR]), and BMI (mean ± SD). Key results are presented as relative changes (↓ decrease, ↑ increase, ↔ no change) compared with uninterrupted sitting.

### 3.3 Primary outcomes

**Figure 2:**
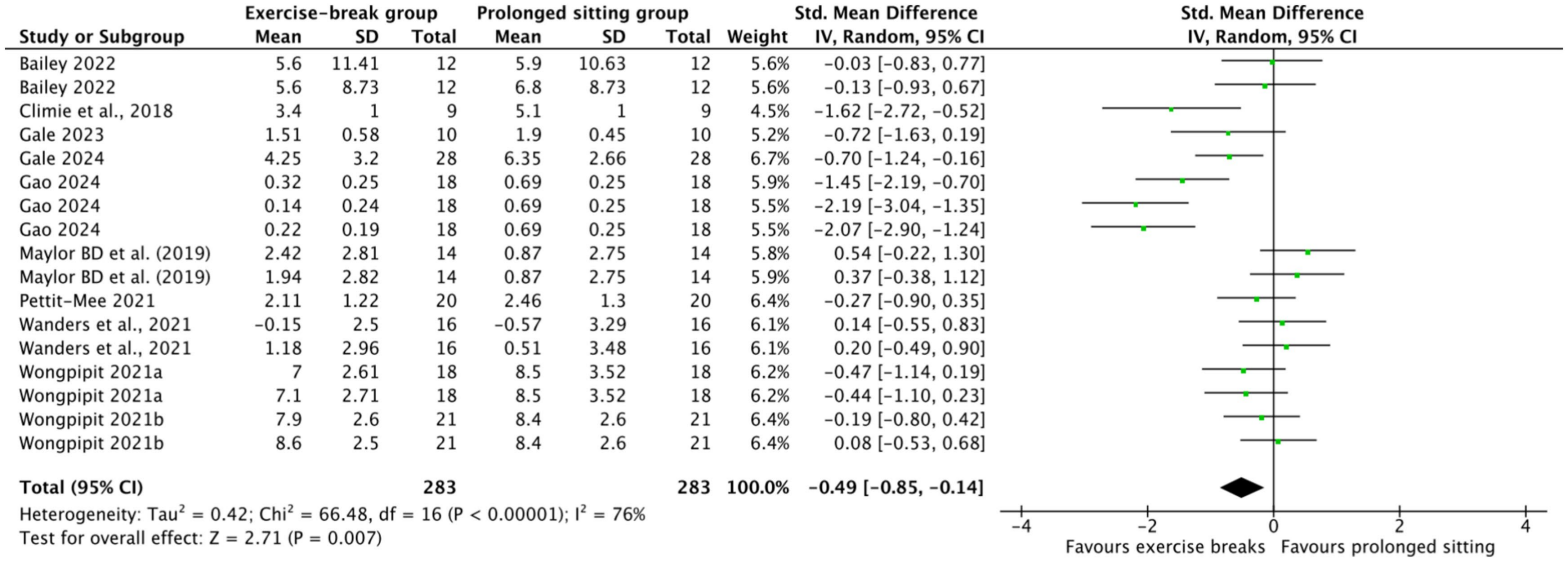
Forest plot of glucose iAUC comparing exercise breaks with prolonged sitting.

**Figure 3:**
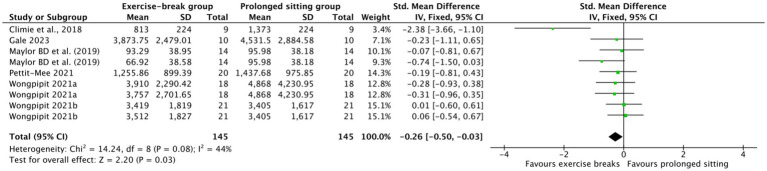
Forest plot of insulin iAUC comparing exercise breaks with prolonged sitting.

Compared with uninterrupted sitting, exercise snacks significantly improved postprandial glucose and insulin dynamics. For glucose, pooled analysis of incremental area under the curve (iAUC) showed a moderate and significant reduction (SMD = −0.49, 95% CI –0.85 to −0.14, *p* = 0.007) (Figure 2), although heterogeneity was substantial (I^2^ = 76%). This highlights the acute efficacy of interrupting prolonged sitting in attenuating postprandial glycemic excursions in obese adults. Similarly, insulin iAUC was significantly reduced in the intervention group (SMD = −0.26, 95% CI –0.50 to −0.03, *p* = 0.03) (Figure 3), with moderate heterogeneity (I^2^ = 44%). These findings support exercise snacks as an effective strategy to blunt both postprandial glycemia and insulinemia, with glucose iAUC emerging as the most consistent indicator. To assess the robustness of these findings, we conducted a sensitivity analysis by excluding studies that involved participants with BMI values near the diagnostic cutoffs. The results remained consistent, with no substantial changes in effect sizes, suggesting that the inclusion of these borderline cases did not significantly affect the overall conclusions.

### 3.4 Secondary outcomes

**Figure 4:**
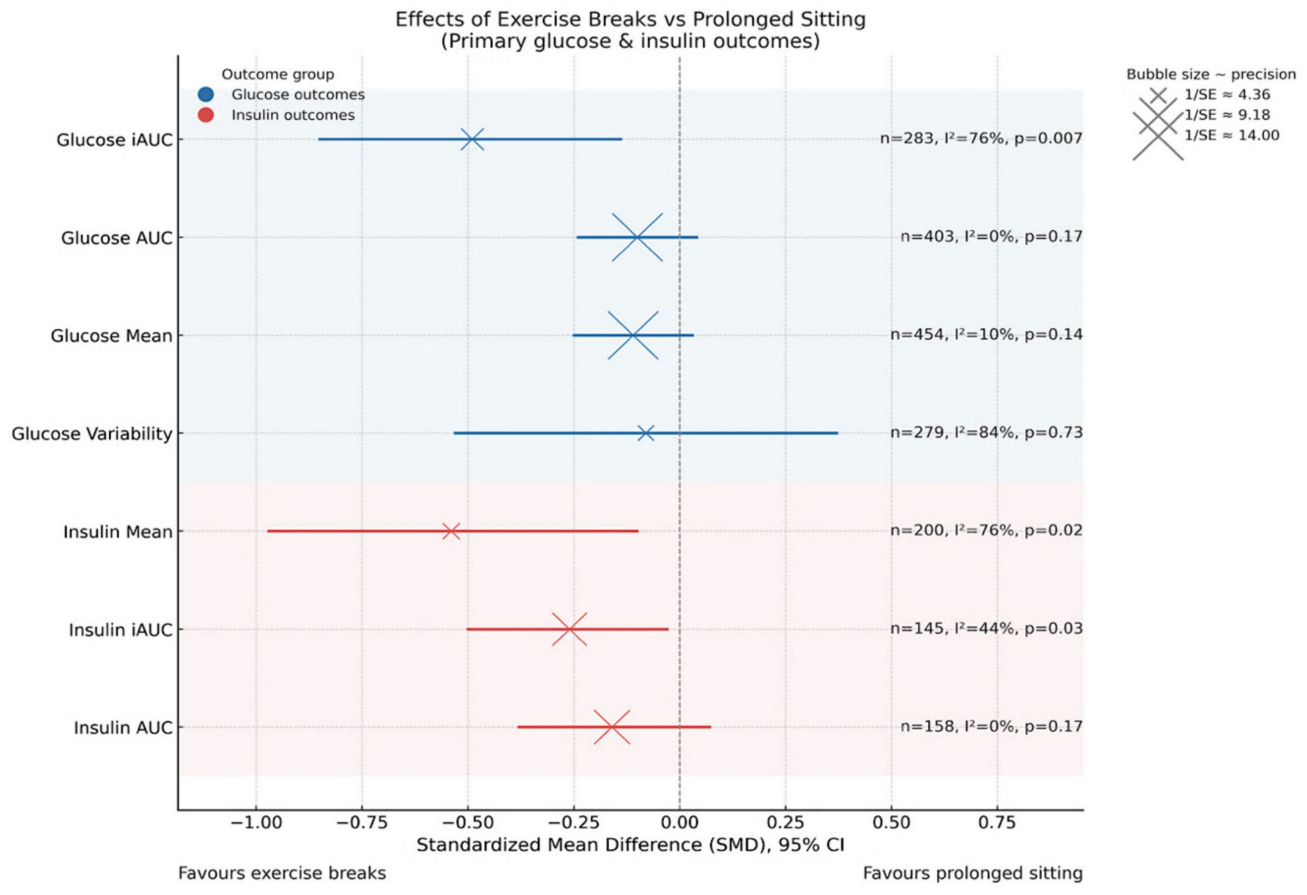
Pooled effects of exercise breaks versus prolonged sitting on glucose and insulin outcomes.

Secondary outcomes showed more heterogeneous and less consistent effects. For glucose, neither total AUC (SMD = −0.10, 95% CI –0.24 to 0.04, *p* = 0.17; I^2^ = 0%) nor mean glucose (SMD = −0.11, 95% CI –0.25 to 0.03, *p* = 0.14; I^2^ = 10%) demonstrated significant improvements, though both pointed toward a modest lowering effect. For insulin, mean levels were significantly reduced (SMD = −0.54, 95% CI –0.97 to −0.10, *p* = 0.02), albeit with high heterogeneity (I^2^ = 76%), whereas total AUC showed only a non-significant downward trend (SMD = −0.16, 95% CI –0.38 to 0.07, *p* = 0.17; I^2^ = 0%). Collectively, these results suggest that while exercise snacks consistently improve postprandial excursions (iAUC), their influence on mean or total exposure metrics is more variable and warrants further confirmation in larger, standardized trials. A summary plot of all glucose and insulin outcomes is presented in Figure 4, while detailed forest plots for each outcome are provided in Supplementary Figures S1–S5.

### 3.5 Subgroup analysis

To further explore potential effect modifiers, pre-specified subgroup analyses were conducted according to participant characteristics (sex, age, BMI) and intervention features (activity type, break frequency, bout duration, total daily dose, Intervention Intensity). Given their clinical relevance and data availability, detailed subgroup results for glucose iAUC and insulin iAUC are presented in the main text (Tables 2, 3), while subgroup analyses for other secondary outcomes are presented in the Supplementary Table S2: subgroup analyses for glucose AUC outcomes; Supplementary Table S3: subgroup analyses for mean glucose outcomes; Supplementary Table S4: subgroup analyses for glucose variability indices outcomes; Supplementary Table S5: subgroup analyses for insulin AUC outcomes; and Supplementary Table S6: subgroup analyses for mean insulin outcomes.

**Table 2:** Subgroup analyses for glucose iAUC outcomes.

Subgroup	k (*N*)	SMD (95% CI)	*P*-value	I^2^ (%)	P_b_
Sex					0.004
Male	132	−0.92 [−1.57, −0.28]	0.005	84%	
Female	28	0.46 [−0.08, 0.99]	0.09	0%	
Mixed	123	−0.33 [−0.68, 0.03]	0.07	45%	
Age					0.007
Young adults	170	−0.87 [−1.36, −0.37]	0.0006	78%	
Middle-aged and older adults	113	−0.02 [−0.38, 0.34]	0.9	44%	
BMI					0.32
Mild obesity	229	−0.56 [−1.02, −0.11]	0.01	81%	
Moderate-to-severe obesity	54	−0.26 [−0.64, 0.12]	0.18	0%	
Intervention type					0.002
Standing	24	−0.08 [−0.65, 0.49]	0.78	0%	
Walking	114	−0.74 [−1.37, −0.11]	0.02	80%	
Resistance exercise	65	−1.23 [−1.94, −0.53]	0.0006	66%	
Cycling	32	0.17 [−0.32, 0.66]	0.49	0%	
Running	28	0.46 [−0.08, 0.99]	0.09	0%	
Leg fidgeting	20	−0.27 [−0.90, 0.35]	0.39	–	
Break frequency					0.17
High frequency	194	−0.27 [−0.53, −0.00]	0.05	38%	
Low frequency	89	−1.03 [−2.09, 0.02]	0.05	90%	
Bout duration					0.05
Short duration (≤3 min)	174	−0.77 [−1.26, −0.28]	0.002	78%	
Long duration (>3 min)	109	−0.11 [−0.55, 0.33]	0.63	62%	
Total daily dose					0.02
Low dose (≤30 min/day)	72	−1.51 [−2.34, −0.68]	0.0004	79%	
Moderate-low dose (31–60 min/day)	158	−0.10 [−0.39, 0.20]	0.52	41%	
Moderate-high dose (61–120 min/day)	33	−0.52 [−1.41, 0.37]	0.25	67%	
High dose (>120 min/day)	20	−0.27 [−0.90, 0.35]	0.39	–	

**Table 3:** Subgroup analyses for insulin iAUC outcomes.

Subgroup	k (*N*)	SMD (95% CI)	*P*-value	I^2^ (%)	P_b_
Sex					0.36
Male	28	−0.39 [−0.92, 0.14]	0.15	33%	
Female	78	−0.11 [−0.43, 0.20]	0.48	0%	
Mixed	39	−0.50 [−0.97, −0.03]	0.04	79%	
Age					0.14
Young adults	88	−0.13 [−0.42, 0.17]	0.4	0%	
Middle-aged and older adults	57	−0.49 [−0.88, −0.11]	0.01	73%	
BMI					0.8
Mild obesity	115	−0.28 [−0.55, −0.02]	0.04	58%	
Moderate-to-severe obesity	30	−0.20 [−0.71, 0.30]	0.43	0%	
Intervention type					0.23
Walking	78	−0.11 [−0.43, 0.20]	0.48	0%	
Resistance exercise	19	−0.93 [−1.65, −0.20]	0.01	86%	
Running	28	−0.39 [−0.92, 0.14]	0.15	33%	
Leg fidgeting	20	−0.19 [−0.81, 0.43]	0.55	–	
Break frequency					0.28
High frequency	92	−0.37 [−0.66, −0.07]	0.02	60%	
Low frequency	53	−0.10 [−0.48, 0.28]	0.62	0%	
Bout duration					0.28
Short duration (≤3 min)	92	−0.37 [−0.66, −0.07]	0.02	60%	
Long duration (>3 min)	53	−0.10 [−0.48, 0.28]	0.62	0%	
Total daily dose					0.004
Moderate–low dose (31–60 min/day)	116	−0.19 [−0.45, 0.07]	0.15	0%	
Moderate–high dose (61–120 min/day)	9	−2.38 [−3.66, −1.10]	0.0003	–	
High dose (>120 min/day)	20	−0.19 [−0.81, 0.43]	0.55	–	

#### 3.5.1 Sex

For glucose iAUC, men showed significant reductions (SMD = −0.92, 95% CI –1.57 to −0.28, *p* = 0.005), whereas women exhibited a non-significant increase (SMD = 0.46, *p* = 0.09). Mixed-sex groups demonstrated a borderline reduction (SMD = −0.34, *p* = 0.05), with a significant subgroup difference (*p* = 0.004). Insulin iAUC was significantly reduced only in mixed-sex groups (SMD = −0.50, *p* = 0.04), with no significant differences between sexes (*p* = 0.36).

#### 3.5.2 Age

Among younger adults (<30 years), glucose tAUC (SMD = −0.21, *p* = 0.04) and mean glucose (SMD = −0.23, *p* = 0.03) were significantly reduced, while no effects were observed in older adults (≥30 years). Glucose variability increased in younger groups (SMD = 0.37, *p* = 0.0005) but decreased in older adults (SMD = −1.58, *p* = 0.02), with significant subgroup differences (*p* = 0.006). For insulin iAUC, significant reductions were observed in older adults (SMD = −0.49, *p* = 0.01), whereas younger adults showed no effect; subgroup differences were not significant (*p* = 0.14).

#### 3.5.3 BMI

No significant effects were found for glucose outcomes when stratified by BMI (<32 vs. ≥32 kg/m^2^). Insulin iAUC was significantly reduced in the mildly obese group (SMD = −0.28, *p* = 0.04), but not in the moderate-to-severe obese group (*p* = 0.43); subgroup differences were not significant (*p* = 0.80).

#### 3.5.4 Intervention type

Walking and resistance exercise yielded the most consistent benefits. Walking reduced glucose iAUC (SMD = −0.74, *p* = 0.02), while resistance exercise showed significant effects on glucose iAUC (SMD = −1.23, *p* < 0.001), insulin iAUC (SMD = −0.93, *p* = 0.01), and glucose tAUC (SMD = −0.29, *p* = 0.01). Standing and resistance exercise both showed borderline improvements in mean glucose (*p* = 0.05). Cycling, running, and leg fidgeting showed no significant effects.

#### 3.5.5 Break frequency

High-frequency breaks (≤30 min) significantly reduced insulin iAUC (SMD = −0.37, *p* = 0.02) and glucose tAUC (SMD = −0.18, *p* = 0.03), with a borderline reduction in glucose iAUC (*p* = 0.05). Low-frequency breaks (>30 min) showed a borderline reduction in mean glucose (SMD = −0.40, *p* = 0.05), but no other significant effects. Subgroup differences were not significant (*p* = 0.17).

#### 3.5.6 Bout duration

Short bouts (≤3 min) significantly improved glucose iAUC (SMD = −0.77, *p* = 0.002), insulin iAUC (SMD = −0.37, *p* = 0.02), and glucose tAUC (SMD = −0.18, *p* = 0.03). Longer bouts (>3 min) were not effective. Subgroup differences for glucose iAUC were borderline significant (*p* = 0.05).

#### 3.5.7 Total daily dose

For glucose iAUC, the low-dose group (≤30 min/day) demonstrated the largest effect (SMD = −1.51, *p* = 0.0004), whereas higher doses did not yield stronger benefits (P_difference = 0.02). For insulin iAUC, the moderate-high dose group (61–120 min/day) showed the greatest reduction (SMD = −2.38, *p* = 0.0003), while other dose groups were not significant (P_difference = 0.004). No consistent effects were observed for glucose mean or insulin mean/AUC.

### 3.6 Meta-regression analysis and sensitivity analysis

**Figure 5:**
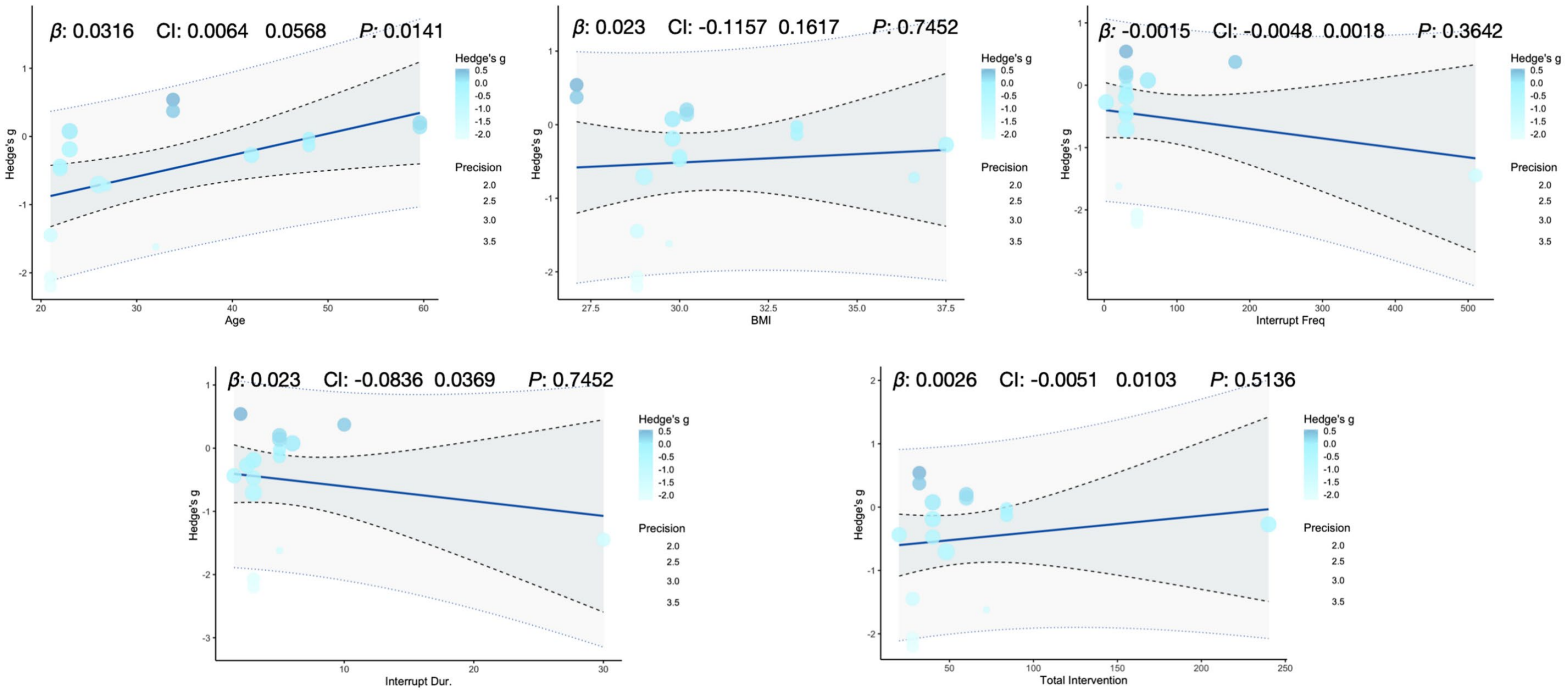
Meta-regression analysis of key variables affecting glucose iAUC.

**Figure 6:**
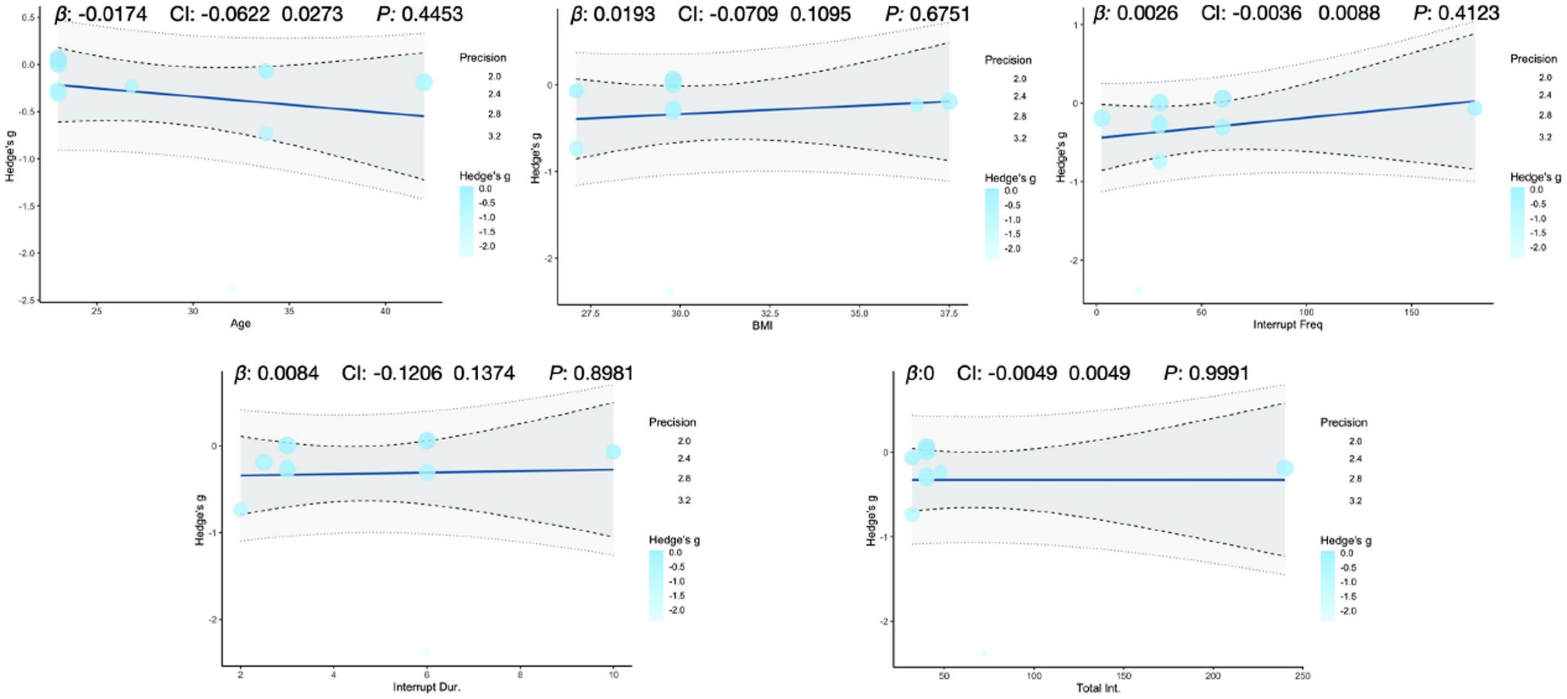
Meta-regression analysis of key variables affecting insulin iAUC.

**Figure 7:**
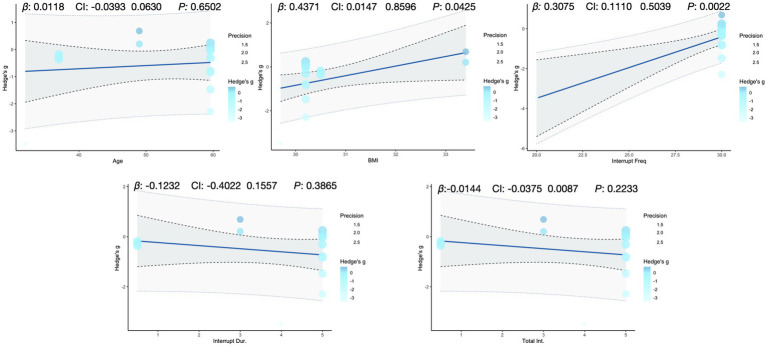
Meta-regression analysis of key variables affecting insulin mean.

To explore potential sources of heterogeneity, we ran meta-regressions linking two primary outcomes (glucose iAUC, insulin iAUC) and one secondary outcome (mean insulin) to five continuous moderators (age, BMI, interruption frequency, single-bout duration, and total intervention duration; Figures 5–7). Age was significantly associated with higher glucose iAUC (*p* = 0.014). No moderator reached significance for insulin iAUC. For mean insulin, both BMI (*p* = 0.043) and interruption frequency (*p* = 0.002) were significant predictors. Single-bout duration and total intervention duration were not significantly associated with any outcome. Across models, R^2^ values were generally low, indicating limited explanatory power.

**Figure 8:**
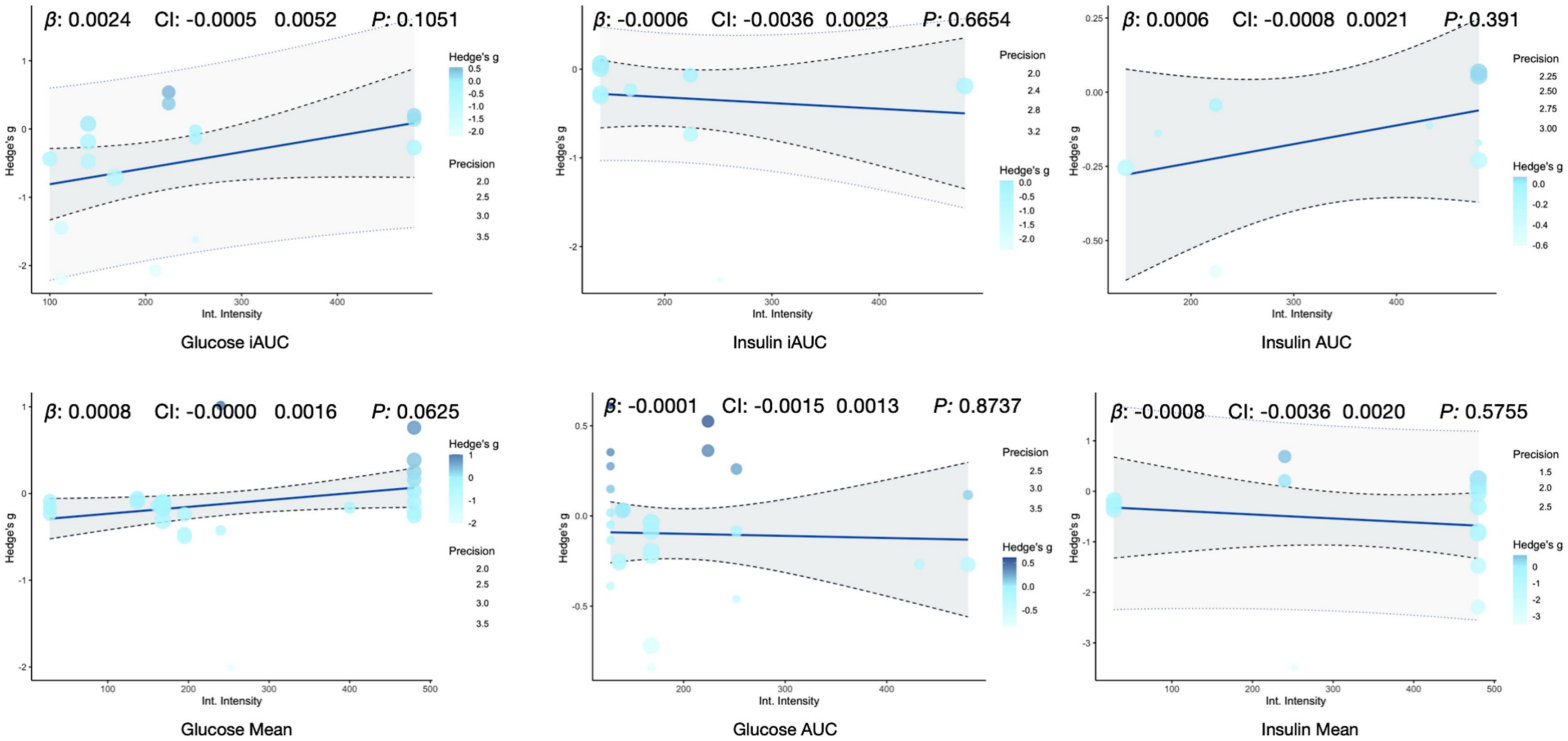
Meta-regression analysis of intervention intensity and its effect on outcome indicators.

Given the significant impact of exercise intensity on insulin sensitivity (55), we standardized and quantified the exercise intensity in the studies. Using the Adult Physical Activity Questionnaire (56), we converted the intervention data from each study into daily exercise intensity. To explore the effect of exercise intensity on the primary outcomes, we conducted meta-regression analysis, treating exercise intensity as a continuous variable. The results of these analyses are shown in Figure 8. The findings indicate that the effect of exercise intensity on the outcomes is minimal, with most effects being statistically insignificant.

To examine the robustness of the pooled results, sensitivity analyses were conducted using the leave-one-out method. Excluding each study in turn did not materially alter the pooled effect estimates or their 95% confidence intervals, suggesting that the overall conclusions were not driven by any single study and were stable.

### 3.7 Risk of bias assessment and publication bias

**Figure 9:**
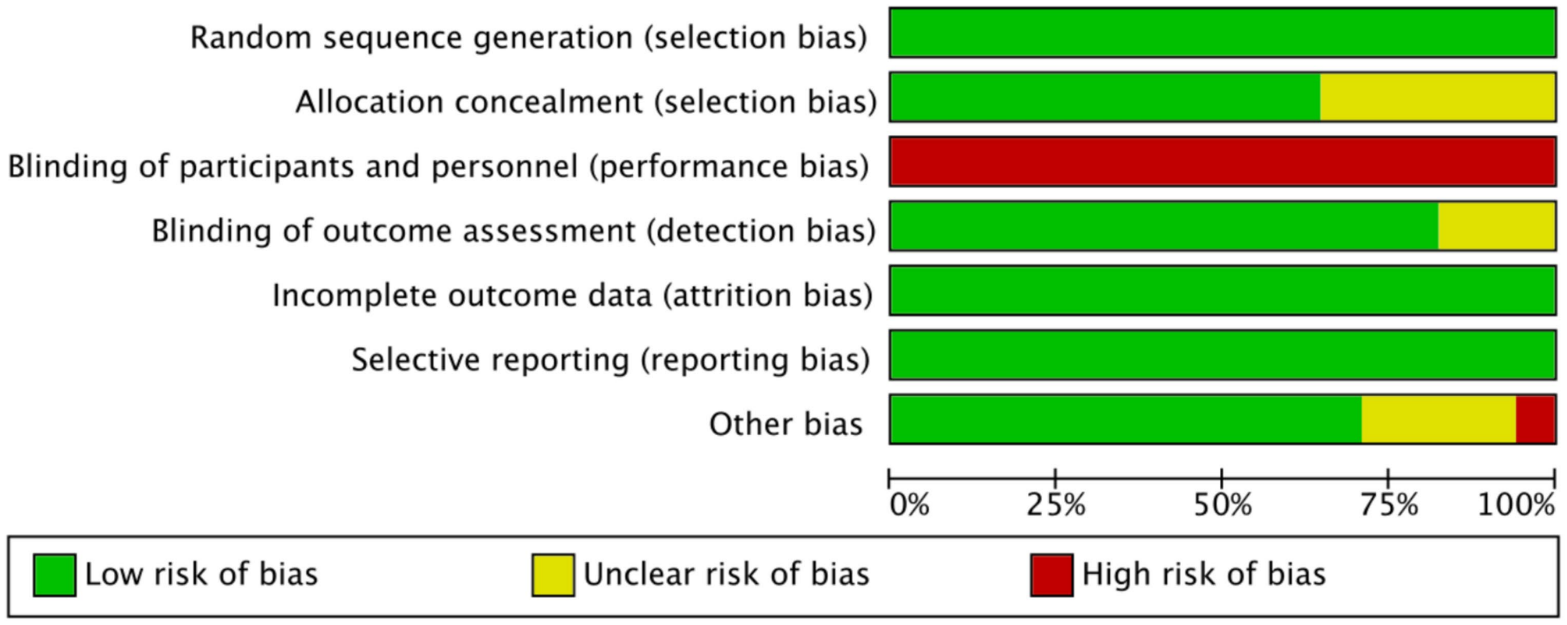
Risk of bias assessment across included studies (Cochrane risk of bias tool).

The methodological quality of the 17 included studies was assessed using the Cochrane Collaboration’s Risk of Bias tool (Figure 9). Most trials were judged to have a low risk of bias in domains such as random sequence generation, completeness of outcome data, and selective reporting, indicating overall methodological robustness. However, allocation concealment was often insufficiently reported, leading to several ratings of unclear risk. Blinding of participants and personnel was consistently judged at high risk due to the inherently visible nature of the interventions (e.g., exercise vs. uninterrupted sitting), although blinding of outcome assessment was generally well implemented, with only a few studies rated as unclear. A small number of studies raised concerns under the “other bias” domain, such as limited sample sizes or incomplete reporting. Overall, the risk of bias across studies was moderate to low, with the primary limitation being the unavoidable lack of blinding of participants and intervention providers.

To assess publication bias, glucose iAUC and glucose AUC, each with ≥10 studies, were formally tested. Begg’s test indicated no evidence of publication bias (iAUC: *p* = 0.655; AUC: *p* = 0.655), and Egger’s regression also showed no small-study effects (iAUC: *p* = 0.484; AUC: *p* = 0.890) (Table 4). These findings suggest that the pooled estimates for glucose-related outcomes are unlikely to be substantially influenced by publication bias. For insulin iAUC and other secondary outcomes, the number of studies was fewer than 10, so Begg’s and Egger’s tests were not performed. Instead, funnel plots were inspected qualitatively, and no obvious asymmetry was detected.

**Table 4:** Publication bias assessment for glucose-related outcomes using Begg’s and Egger’s tests.

Outcome	Begg’s test Z	Begg’s test (*P*)	Egger’s regression (*P*)
Glucose iAUC	−0.45	0.655	0.484
Glucose AUC	−0.45*	0.655	0.890

*Identical Begg’s Z values were returned for iAUC and AUC in the Stata output. *P* < 0.05 indicates potential publication bias.

### 3.8 GRADE evaluation of evidence quality

The quality of evidence for each primary outcome was assessed using the GRADE methodology, with outcomes rated as high, moderate, low, or very low. Glucose iAUC and Insulin iAUC were rated as moderate due to concerns about inconsistency. Other outcomes, including Glucose AUC, Insulin AUC, and Insulin Mean, were rated as low due to imprecision and wide confidence intervals. Detailed GRADE ratings for each outcome are summarized in Table 5.

**Table 5:** GRADE assessment of primary outcomes.

Outcome	No of participants (studies)	Certainty assessment					Standardized mean effect (95% CI)	GRADE*
		Risk of bias	Inconsistency	Indirectness	Imprecision	Publication bias		
Glucose iAUC	283 (10 RCTs)	Moderate	Serious	Not serious	Not serious	None	−0.49 [−0.85, −0.14]	⊕⊕⊕Ο Moderate
Glucose AUC	403 (10 RCTs)	Moderate	Not serious	Not serious	Serious	None	−0.10 [−0.24, 0.04]	⊕⊕ΟΟ Low
Glucose mean	454 (8 RCTs)	Moderate	Not serious	Not serious	Serious	N/A	−0.11 [−0.25, 0.03]	⊕⊕ΟΟ Low
Insulin iAUC	145 (6 RCTs)	Moderate	Serious	Not serious	Not serious	N/A	−0.26 [−0.50, −0.03]	⊕⊕⊕Ο Moderate
Insulin AUC	158 (8 RCTs)	Moderate	Not serious	Not serious	Serious	N/A	−0.16 [−0.38, 0.07]	⊕⊕ΟΟ Low
Insulin mean	200 (4 RCTs)	Moderate	Serious	Not serious	Serious	N/A	−0.54 [−0.97, −0.10]	⊕⊕ΟΟ Low

Certainty of evidence according to Grading of Recommendations, Assessment, Development, and Evaluations (GRADE): High: We are very confident in the estimated effect. The research design is strong, and the results are consistent and precise. We have little to no doubt about the effect’s reliability. Moderate: Our confidence in the estimated effect is moderate. There may be some concerns regarding study quality, consistency, or precision, but the evidence still supports the estimated effect. Low: We have limited confidence in the estimated effect. The evidence has some major limitations (e.g., high risk of bias, inconsistent results, imprecise estimates), and the true effect could be substantially different. Very Low: We have very little confidence in the estimated effect. The evidence has serious limitations, and the actual effect may differ substantially from the estimated effect. No of participants: Total number of participants with pooled effects.

## 4 Discussion

### 4.1 Main findings

This systematic review and meta-analysis demonstrates that interrupting prolonged sitting with brief bouts of physical activity meaningfully improves glycemic and insulinemic responses in adults with obesity. Compared with uninterrupted sitting, activity breaks lowered postprandial glucose and insulin, with glucose outcomes showing a moderate standardized effect and insulin outcomes also clearly favorable. In our pooled analyses, exercise interruptions reduced glucose incremental area under the curve (iAUC) and peak responses, paralleled by decreases in insulin curves. These findings are consistent with Loh et al. (20), who reported moderate reductions in both glucose and insulin (SMD ≈ −0.5) with sitting interruptions. Notably, our work focuses on metabolically healthy adults with obesity, among whom benefits may be greater, aligning with prior evidence that higher BMI is associated with larger improvements from breaking up sitting (20). Collectively, our results confirm the acute metabolic advantages of “exercise snacks” in people with obesity and strengthen the rationale for embedding regular movement into prolonged sitting periods.

### 4.2 Interpreting differences across outcomes

Heterogeneity in outcome definitions (e.g., total AUC vs. iAUC; CGM-derived measures vs. intermittent venous sampling) can influence effect estimates. I^2^ values indicate notable heterogeneity in some outcomes, such as glucose iAUC (I^2^ = 76%), suggesting substantial variation across studies. iAUC subtracts the baseline component and may better isolate postprandial excursions, whereas total AUC reflects overall exposure. We prioritized harmonized metrics where possible to enhance comparability. Prior syntheses suggest modest quantitative differences by measurement method (e.g., CGM versus discrete sampling) without altering the qualitative conclusion that interruptions are beneficial (57–59).

In the present review, activity breaks generally reduced postprandial glycemia across iAUC, total AUC, and mean glucose, with effect sizes varying by metric. Insulin outcomes (e.g., insulin AUC, peak insulin, HOMA-IR) trended in the favorable direction as well; iAUC is particularly sensitive to acute changes in postprandial secretion, whereas fasting or total exposure may require longer observation58. However, the high heterogeneity observed (I^2^ = 76%) suggests that the effects of activity breaks may vary across studies due to differences in intervention characteristics (e.g., frequency, duration, mode) and participant characteristics (e.g., age, BMI). This variability underscores the need for cautious interpretation of the results.

A subset of trials assessed glycemic variability (e.g., SD or CV from CGM) (41, 42, 52); interruptions tended to mitigate excessive variability—a clinically relevant signal given links to cardiovascular risk—though current evidence remains limited and should be interpreted cautiously. Overall, convergence of multiple indicators supports the conclusion, while metric-specific differences underscore the need to consider measurement choices and intervention characteristics when interpreting effects.

### 4.3 Synthesis of subgroup findings

We examined effect modifiers to clarify “for whom” and “how” activity breaks work best.

#### 4.3.1 Participant characteristics

Across prespecified subgroups, we observed several directional patterns with generally limited statistical support. For sex, men showed clear reductions in glucose iAUC whereas the female-only subgroup did not; the formal test for subgroup differences was significant for glucose iAUC, while insulin iAUC did not differ by sex, indicating a possible sex-specific effect on glycemia but not insulinemia (60). For age, a differentiated pattern emerged: adults <30 years benefited more on glucose outcomes (e.g., total AUC and mean glucose), whereas adults ≥30 years exhibited greater reductions in insulin iAUC (61–63); most subgroup tests, however, were non-significant, and estimates were imprecise (64–66). For adiposity, within our obese-only sample we did not detect stronger effects at higher BMI. Indeed, the decline in insulin iAUC was more evident in the <32 kg/m^2^ stratum, and the BMI subgroup test was null—findings that likely reflect small subgroup sizes, a restricted BMI range, and coarse categorization (20, 67, 68).

#### 4.3.2 Intervention characteristics

Mode, frequency, bout duration, and total daily dose contributed to variability. Walking and especially resistance-type “snacks” yielded the most consistent benefits; resistance was associated with larger improvements in glucose iAUC and insulin iAUC, whereas standing showed only borderline reductions in mean glucose (69–71). While the mean effect size for resistance training was significant, the stability of this finding remains uncertain due to the high heterogeneity observed (e.g., I^2^ values). The wide variability in effect sizes highlights the need for caution when interpreting these results. Despite the positive trends observed, further research with larger sample sizes and more consistent intervention protocols is required to establish more robust conclusions. Cycling, running, and leg fidgeting did not demonstrate reliable effects, possibly due to differences in exercise intensity or participant compliance, indicating that not all types of physical activity may yield similar benefits. For break frequency, interrupting sitting every 30 min tended to outperform lower frequencies across several outcomes, though the subgroup difference test was non-significant (21). For bout duration, short bouts (≤3 min) were superior to longer bouts for glucose iAUC and insulin iAUC before accounting for total daily volume. However, when stratified by daily volume, this advantage attenuated, underscoring the importance of the cumulative “frequency × duration” effect (72–74). Finally, the dose–response relationship appeared non-linear: the largest reduction in glucose iAUC occurred with ≤30 min/day, whereas insulin iAUC improvements peaked at 61–120 min/day. Higher daily volumes did not confer additional benefit, possibly due to fatigue, adherence issues, or compensatory behaviors. Additionally, the results of the regression analysis indicated that intervention intensity, including factors such as frequency and total intervention duration, had minimal and mostly non-significant effects on the primary outcomes.

### 4.4 Plausible mechanisms

Skeletal muscle contraction is a central mechanism: prolonged sitting suppresses muscle activity and glucose uptake, whereas even brief standing/walking or resistance movements rapidly stimulate GLUT4 translocation and increase insulin-independent glucose disposal (75–78). These effects can acutely lower glucose and transiently enhance insulin sensitivity, explaining observed declines in insulin curves after only minutes of activity. Resistance-type breaks may be especially potent due to greater recruitment of large muscle groups and type II fibers and a post-exercise “afterburn,” consistent with our subgroup signals (79–81). Contraction-induced myokines (e.g., IL-6) can further augment insulin signaling and exert anti-inflammatory effects (70, 82–84), which is pertinent for individuals with obesity who commonly exhibit low-grade inflammation and insulin resistance (85, 86). Repeated interruptions also raise energy expenditure; while each bout is brief, cumulative energy cost may aid weight management and insulin sensitivity over time (23). Some studies report carryover into the nocturnal period (e.g., lower nighttime glucose), suggesting sustained increases in glucose utilization or enhanced insulin action (20, 87, 88). Thus, rapid muscular glucose clearance, improved insulin sensitivity, and anti-inflammatory signaling likely converge to produce the acute metabolic benefits observed—particularly pronounced in obesity.

### 4.5 Comparison with the literature

Our findings accord with prior syntheses. As noted, Loh et al. (20) reported significant improvements in postprandial glucose and insulin and identified larger benefits with higher BMI (21). Reviews by Saunders and Benatti also support beneficial effects, albeit with somewhat smaller pooled estimates (89, 90). For example, Saunders reported effects of approximately −0.36 for glucose and −0.37 for insulin (Cohen’s d) (90), values slightly below ours, potentially due to inclusion of more metabolically healthy participants and methodological differences. The overarching message is consistent: uninterrupted sitting impairs glucose regulation, and interspersed movement mitigates this harm (91). With respect to implementation details, evidence has been less definitive about “how often.” Yin et al. (21) addressed this gap by recommending breaks at least every 30 minutes; our results align and emphasize the importance of this cadence in adults with obesity. Earlier guidance and reviews often refrained from specifying frequency due to insufficient evidence (92); our estimates help inform that debate.

### 4.6 Practical and policy implications

For adults with obesity or high cardiometabolic risk who sit for long periods (e.g., desk-based workers), we recommend rising at ~30-min intervals for 2–5 min of light walking, standing with simple body-weight resistance, or similar activities. These “exercise snacks” are feasible within daily routines and can meaningfully blunt postprandial glycemic excursions, supporting diabetes prevention. However, it is important to note that the evidence supporting this recommendation is moderate in quality and primarily based on short-term studies with varying methodologies and populations. While the effects on glycemia and insulin sensitivity are generally positive, further research is needed to confirm the applicability and effectiveness of these breaks, especially in specific populations such as older adults and individuals with diabetes.

Clinicians should integrate the recommendation to reduce uninterrupted sitting and add frequent activity breaks into lifestyle counseling, alongside conventional exercise prescriptions (e.g., ≥150 min/week of moderate-intensity exercise). However, clinicians should be aware of the current limitations in evidence, particularly for specific subgroups. At the policy level, workplaces and schools could facilitate standing desks, scheduled short breaks, and education campaigns, consistent with the 2020 WHO Guidelines, which emphasize limiting sedentary time and accumulating physical activity across intensities (93). Messaging such as “move for a few minutes every 30 min of sitting”, aided by wearables or smartphone prompts, may enhance adherence and yield population-level benefits. Given the low cost and scalability (92), promoting sitting interruptions could be a cost-effective prevention strategy in settings with high obesity and diabetes burden, but the generalizability of this recommendation across different groups still requires further validation.

### 4.7 Strengths and limitations

Strengths include adherence to PRISMA, a comprehensive and up-to-date search, and an *a priori* focus on adults with obesity—addressing a gap in prior reviews that largely pooled mixed populations. We conducted extensive subgroup and sensitivity analyses (sex, age, BMI, frequency, mode) and evaluated risk of bias and certainty, finding “low” to “moderate” certainty for key outcomes yet overall robust conclusions. We also explicitly contrasted outcome definitions (iAUC, total AUC, mean), improving methodological transparency.

Limitations include potential publication bias due to restriction to peer-reviewed English-language studies; although funnel plots and Egger tests did not suggest small-study effects, undetected bias remains possible. Heterogeneity in intervention protocols (e.g., frequency, intensity) and measurement (CGM vs. laboratory sampling) persisted despite random-effects modeling and subgroup exploration. Most trials examined acute responses over a single day; thus, our conclusions primarily address short-term effects. Whether sustained interruptions improve longer-term endpoints (e.g., HbA1c) is unknown due to limited long-duration RCTs. Sample sizes were small (often 10–30 participants), and generalizability beyond primarily White and Asian younger/middle-aged adults is uncertain. Performance blinding is inherently infeasible, and control conditions (e.g., dietary standardization) varied. Although both laboratory and free-living settings were eligible, the evidence base was predominantly laboratory-based; only a few free-living studies contributed data (49), and their precision was limited. While the direction of effects in free-living contexts appears broadly consistent, external validity should be interpreted with caution.

### 4.8 Directions for future research

Future work should move beyond acute, laboratory crossovers to adequately powered randomized trials in free-living settings that test durability of effects on fasting glucose, HbA1c, body composition, and diabetes incidence. Head-to-head, factorial, or adaptive designs comparing interruption cadence (e.g., ≤30 vs. > 30 min), bout duration (e.g., 1–2 vs. ~ 5 min), mode (walking, resistance, cycling, stretching) and intensity are needed to define minimal effective doses and optimal combinations, rather than inferring from single-schedule studies (e.g., ~30-min cadence). Parallel mechanistic endpoints—such as skeletal-muscle glucose uptake, GLUT4 content/translocation, and inflammatory mediators—would clarify pathways and dose thresholds. Recruitment should extend to older adults with obesity, people with diabetes/metabolic syndrome, and highly sedentary occupations; larger samples are required to examine sex differences and inter-individual variability (including phenotypic or genetic moderators), where current evidence remains inconclusive. Pragmatic trials leveraging wearables, app-based prompts, and simple incentives can address adherence, feasibility, and durability under real-world conditions. Finally, multicomponent strategies that pair sitting interruptions with dietary modification or structured exercise, and assessment of broader outcomes—cardiovascular, cognitive, mental-health, and cost-effectiveness—will be essential to translate acute benefits into quantitative guidance on how often, how long, and what to do for adults with obesity.

## 5 Conclusion

This systematic review and meta-analysis demonstrates that interrupting prolonged sitting with brief “exercise snacks” may significantly improve glucose and insulin regulation in adults with obesity. Compared with uninterrupted sitting, engaging in 2–5 min of activity approximately every 30 min resulted in reductions in postprandial glucose iAUC and insulin responses, with walking and resistance exercises generally showing more consistent benefits, while standing provided borderline improvements. Subgroup analyses indicated that intervention effects were jointly moderated by participant characteristics and protocol design: men and younger adults exhibited greater improvements in glycemic outcomes, whereas middle-aged and older adults benefited more in terms of reduced insulin responses. Individuals with mild obesity showed stronger improvements in insulin metabolism, although no clear BMI-stratified differences were observed for glucose outcomes. Regarding intervention features, high-frequency (≤30 min) and short-bout (≤3 min) interruptions were superior to less frequent or longer bouts, and dose–response analyses suggested a non-linear pattern, with low-to-moderate daily volumes (≤30–120 min/day) being more advantageous than higher doses. Overall, these findings support the recommendation that adults with obesity should incorporate regular breaks—every ≤30 min for 2–5 min, primarily consisting of walking or simple resistance activities—into daily life and clinical management. However, due to the observed heterogeneity and the non-significant effects of intensity, we urge caution when implementing these recommendations in diverse populations. Further long-term trials are warranted to confirm the sustainability of these effects and to determine optimal intervention parameters.

## Data Availability

The original contributions presented in the study are included in the article/Supplementary material, further inquiries can be directed to the corresponding author.
