# Supplementary material for: Acute effects of exercise snacks on postprandial glucose and insulin metabolism in adults with obesity: a systematic review and meta-analysis
Source: Front Nutr. 2025 Nov 20;12:1708301. doi: 10.3389/fnut.2025.1708301 (PMC12677009; doi:10.3389/fnut.2025.1708301)
Supplement: Supplementary file 1 [file Table_1.docx]

Table S1. Full search strategy for Cochrane Library (as of July 10, 2025)

| **Step** | **Search terms** |
| --- | --- |
| #1 | MeSH descriptor: [Adult] explode all trees |
| #2 | (adult* OR healthy adult*):ti,ab,kw |
| #3 | #1 OR #2 |
| #4 | MeSH descriptor: [Obesity] explode all trees |
| #5 | (obes* OR abdominal obes* OR "body mass index" OR BMI):ti,ab,kw |
| #6 | #4 OR #5 |
| #7 | MeSH descriptor: [Sedentary Behavior] explode all trees |
| #8 | (sedentar* OR "sedentary lifestyle" OR physical inactiv* OR "lack of physical activity" OR "sedentary time*" OR "time sedentary"):ti,ab,kw |
| #9 | #7 OR #8 |
| #10 | #3 AND #6 AND #9 |
| #11 | ("sedentary breaks" OR "activity breaks" OR "interrupt sitting" OR "breaking up sitting" OR "sit-stand" OR microbouts OR "exercise snacks" OR "intermittent activity" OR "accumulated physical activity" OR "short bouts of activity" OR "movement breaks" OR "frequent movement breaks" OR break* OR interrupt* OR fragment* OR fraction* OR disrupt*):ti,ab,kw |
| #12 | MeSH descriptor: [Blood Glucose] explode all trees |
| #13 | ("glycemic variability" OR "glycaemic variability" OR "glucose variability" OR "glucose fluctuation*" OR MAGE OR "mean amplitude of glycemic excursions" OR "standard deviation of glucose" OR "CV glucose" OR "blood glucose excursion" OR "glucose excursions" OR "time in range" OR TIR OR "continuous glucose monitoring" OR CGM OR "blood glucose" OR "glucose blood" OR "blood sugar" OR "sugar blood"):ti,ab,kw |
| #14 | #12 OR #13 |
| #15 | #10 AND #11 AND #14 |
