# Supplementary material for: Acute effects of exercise snacks on postprandial glucose and insulin metabolism in adults with obesity: a systematic review and meta-analysis
Source: Front Nutr. 2025 Nov 20;12:1708301. doi: 10.3389/fnut.2025.1708301 (PMC12677009; doi:10.3389/fnut.2025.1708301)
Supplement: Supplementary file 2 [file Table_2.docx]

**Table S2.** Subgroup analyses for glucose AUC outcomes

| **Subgroup** | **k (N)** | **SMD  (95% CI)** | **P-value** | **I² (%)** | **P_b_** |
| --- | --- | --- | --- | --- | --- |
| Sex |  |  |  |  | 0.08 |
| Female | 28 | 0.44 [–0.09, 0.97] | 0.1 | 0% |  |
| Male | 62 | 0.00 [–0.35, 0.35] | 0.99 | 0% |  |
| Mixed | 313 | –0.17 [–0.32, –0.01] | 0.04 | 0% |  |
| Age |  |  |  |  | 0.13 |
| Young adults | 192 | –0.21 [–0.41, –0.01] | 0.04 | 1% |  |
| Middle-aged and older adults | 211 | 0.00 [–0.19, 0.20] | 0.96 | 0% |  |
| BMI |  |  |  |  | 0.33 |
| Mild obesity | 311 | –0.06 [–0.22, 0.10] | 0.45 | 0% |  |
| Moderate-to-severe obesity | 92 | –0.23 [–0.52, 0.07] | 0.13 | 0% |  |
| **Intervention Type** |  |  |  |  | 0.12 |
| Standing | 44 | 0.01 [–0.41, 0.43] | 0.96 | 0% |  |
| Walking | 80 | –0.10 [–0.41, 0.21] | 0.51 | 0% |  |
| Resistance exercise | 159 | –0.29 [–0.51, –0.07] | 0.01 | 0% |  |
| Stair climbing | 72 | 0.10 [–0.23, 0.43] | 0.54 | 0% |  |
| Running | 28 | 0.44 [–0.09, 0.97] | 0.1 | 0% |  |
| Leg fidgeting | 20 | –0.27 [–0.89, 0.35] | 0.4 | – |  |
| **Break Frequency** |  |  |  |  | 0.06 |
| High frequency | 296 | –0.18 [–0.34, –0.02] | 0.03 | 0% |  |
| Low frequency | 107 | 0.12 [–0.15, 0.39] | 0.38 | 0% |  |
| **Bout Duration** |  |  |  |  | 0.05 |
| Short duration (≤3 min) | 298 | –0.18 [–0.34, –0.02] | 0.03 | 0% |  |
| Long duration (>3 min) | 105 | 0.14 [–0.13, 0.41] | 0.32 | 0% |  |
| **Total Daily Dose** |  |  |  |  | 0.62 |
| Low dose（≤30 min/day） | 72 | 0.10 [–0.23, 0.43] | 0.54 | 0% |  |
| Moderate-low dose（31–60 min/day） | 258 | –0.15 [–0.33, 0.04] | 0.13 | 12% |  |
| Moderate-high dose（61–120 min/day） | 33 | –0.06 [–0.55, 0.43] | 0.81 | 0% |  |
| High dose（>120 min/day） | 40 | –0.17 [–0.61, 0.27] | 0.44 | 0% |  |
