# Supplementary material for: Acute effects of exercise snacks on postprandial glucose and insulin metabolism in adults with obesity: a systematic review and meta-analysis
Source: Front Nutr. 2025 Nov 20;12:1708301. doi: 10.3389/fnut.2025.1708301 (PMC12677009; doi:10.3389/fnut.2025.1708301)
Supplement: Supplementary file 3 [file Table_3.docx]

**Table S3.** Subgroup analyses for mean glucose outcomes

| **Subgroup** | **k (N)** | **SMD  (95% CI)** | **P-value** | **I² (%)** | **P_b_** |
| --- | --- | --- | --- | --- | --- |
| Sex |  |  |  |  | **0.88** |
| Female | 20 | –0.15 [–0.77, 0.47] | 0.63 | 0% |  |
| Male | – | – | – | – |  |
| Mixed | 434 | –0.10 [–0.25, 0.05] | 0.17 | 17% |  |
| Age |  |  |  |  | **0.12** |
| Young adults | 191 | –0.23 [–0.43, –0.03] | 0.03 | 0% |  |
| Middle-aged and older adults | 263 | –0.02 [–0.23, 0.18] | 0.82 | 28% |  |
| BMI |  |  |  |  | **0.62** |
| Mild obesity | 380 | –0.12 [–0.28, 0.04] | 0.13 | 18% |  |
| Moderate-to-severe obesity | 74 | –0.03 [–0.36, 0.29] | 0.85 | 0% |  |
| **Intervention Type** |  |  |  |  | **0.08** |
| Standing | 51 | –0.40 [–0.79, –0.01] | 0.05 | 0% |  |
| Walking | 74 | –0.03 [–0.36, 0.29] | 0.85 | 0% |  |
| Resistance exercise | 191 | –0.23 [–0.45, –0.00] | 0.05 | 16% |  |
| Cycling | 138 | 0.12 [–0.12, 0.36] | 0.33 | 0% |  |
| **Break Frequency** |  |  |  |  | **0.12** |
| High frequency | 403 | –0.07 [–0.22, 0.08] | 0.37 | 12% |  |
| Low frequency | 51 | –0.40 [–0.79, –0.01] | 0.05 | 0% |  |
| **Bout Duration** |  |  |  |  | **0.33** |
| Short duration (≤3 min) | 255 | –0.17 [–0.36, 0.02] | 0.08 | 13% |  |
| Long duration (>3 min) | 199 | –0.03 [–0.23, 0.18] | 0.79 | 7% |  |
| **Total Daily Dose** |  |  |  |  | **0.23** |
| Low dose（≤30 min/day） | 42 | –0.16 [–0.59, 0.27] | 0.46 | 0% |  |
| Moderate-low dose（31–60 min/day） | 342 | –0.02 [–0.17, 0.13] | 0.76 | 0% |  |
| Moderate-high dose（61–120 min/day） | 19 | –1.04 [–2.84, 0.76] | 0.25 | 83% |  |
| High dose（>120 min/day） | 51 | –0.40 [–0.79, –0.01] | 0.05 | 0% |  |
