# Supplementary material for: Acute effects of exercise snacks on postprandial glucose and insulin metabolism in adults with obesity: a systematic review and meta-analysis
Source: Front Nutr. 2025 Nov 20;12:1708301. doi: 10.3389/fnut.2025.1708301 (PMC12677009; doi:10.3389/fnut.2025.1708301)
Supplement: Supplementary file 4 [file Table_4.docx]

**Table S4.** Subgroup analyses for glucose variability indices outcomes

| **Subgroup** | **k (N)** | **SMD  (95% CI)** | **P-value** | **I² (%)** | **P_b_** |
| --- | --- | --- | --- | --- | --- |
| Age |  |  |  |  | **0.006** |
| Young adults | 280 | 0.37 [0.16, 0.57] | 0.0005 | 33% |  |
| Middle-aged and older adults | 51 | –1.58 [–2.95, –0.22] | 0.02 | 87% |  |
| BMI |  |  |  |  | **0.85** |
| Mild obesity | 307 | –0.07 [–0.48, 0.35] | 0.75 | 83% |  |
| Moderate-to-severe obesity | 24 | 0.00 [–0.57, 0.57] | 1 | 0% |  |
| **Intervention Type** |  |  |  |  | **0.85** |
| Standing | 24 | 0.00 [–0.57, 0.57] | 1 | 0% |  |
| Resistance exercise | 307 | –0.07 [–0.48, 0.35] | 0.75 | 83% |  |
| **Bout Duration** |  |  |  |  | **0.85** |
| Short duration (≤3 min) | 307 | –0.07 [–0.48, 0.35] | 0.75 | 83% |  |
| Long duration (>3 min) | 24 | 0.00 [–0.57, 0.57] | 1 | 0% |  |
| **Total Daily Dose** |  |  |  |  | **0.23** |
| Moderate–low dose (31–60 min/day) | 280 | 0.37 [0.16, 0.57] | 0.0005 | 33% |  |
| Moderate–high dose (61–120 min/day) | 51 | –1.58 [–2.95, –0.22] | 0.02 | 87% |  |
