# Supplementary material for: Acute effects of exercise snacks on postprandial glucose and insulin metabolism in adults with obesity: a systematic review and meta-analysis
Source: Front Nutr. 2025 Nov 20;12:1708301. doi: 10.3389/fnut.2025.1708301 (PMC12677009; doi:10.3389/fnut.2025.1708301)
Supplement: Supplementary file 5 [file Table_5.docx]

**Table S5.** Subgroup analyses for insulin AUC outcomes

| **Subgroup** | **k (N)** | **SMD  (95% CI)** | **P-value** | **I² (%)** | **P_b_** |
| --- | --- | --- | --- | --- | --- |
| Sex |  |  |  |  | **0.58** |
| Female | 28 | –0.32 [–0.87, 0.23] | 0.26 | 7% |  |
| Male | 20 | –0.14 [–0.76, 0.48] | 0.65 | 0% |  |
| Mixed | 110 | –0.12 [–0.38, 0.15] | 0.38 | 0% |  |
| Age |  |  |  |  | **0.97** |
| Young adults | 10 | –0.14 [–1.02, 0.74] | 0.76 | – |  |
| Middle-aged and older adults | 148 | –0.16 [–0.39, 0.07] | 0.18 | 0% |  |
| BMI |  |  |  |  | **0.57** |
| Mild obesity | 90 | –0.10 [–0.39, 0.19] | 0.51 | 0% |  |
| Moderate-to-severe obesity | 68 | –0.23 [–0.57, 0.11] | 0.18 | 0% |  |
| **Intervention Type** |  |  |  |  | **0.91** |
| Standing | 20 | –0.14 [–0.76, 0.48] | 0.65 | 0% |  |
| Walking | 38 | –0.25 [–0.71, 0.20] | 0.27 | 0% |  |
| Resistance exercise | 10 | –0.14 [–1.02, 0.74] | 0.76 | – |  |
| Cycling | 42 | 0.06 [–0.37, 0.49] | 0.78 | 0% |  |
| Running | 28 | –0.32 [–0.87, 0.23] | 0.26 | 7% |  |
| Leg fidgeting | 20 | –0.23 [–0.85, 0.39] | 0.47 | – |  |
| **Break Frequency** |  |  |  |  | **0.76** |
| High frequency | 144 | –0.17 [–0.40, 0.07] | 0.16 | 0% |  |
| Low frequency | 14 | –0.04 [–0.78, 0.70] | 0.91 | – |  |
| **Bout Duration** |  |  |  |  | **0.2** |
| Short duration (≤3 min) | 92 | –0.28 [–0.57, 0.01] | 0.06 | 0% |  |
| Long duration (>3 min) | 66 | 0.01 [–0.33, 0.35] | 0.94 | 0% |  |
| **Total Daily Dose** |  |  |  |  | **0.88** |
| Moderate–low dose (31–60 min/day) | 118 | –0.15 [–0.40, 0.11] | 0.26 | 0% |  |
| High dose (>120 min/day) | 40 | –0.19 [–0.62, 0.25] | 0.41 | 0% |  |
