# Supplementary material for: Acute effects of exercise snacks on postprandial glucose and insulin metabolism in adults with obesity: a systematic review and meta-analysis
Source: Front Nutr. 2025 Nov 20;12:1708301. doi: 10.3389/fnut.2025.1708301 (PMC12677009; doi:10.3389/fnut.2025.1708301)
Supplement: Supplementary file 6 [file Table_6.docx]

**Table S6.** Subgroup analyses for mean insulin outcomes

| **Subgroup** | **k (N)** | **SMD  (95% CI)** | **P-value** | **I² (%)** | **P_b_** |
| --- | --- | --- | --- | --- | --- |
| BMI |  |  |  |  | **0.009** |
| Mild obesity | 184 | –0.68 [–1.14, –0.22] | 0.004 | 77% |  |
| Moderate-to-severe obesity | 16 | 0.44 [–0.27, 1.15] | 0.22 | 0% |  |
| **Intervention Type** |  |  |  |  | **0.03** |
| Walking | 16 | 0.44 [–0.27, 1.15] | 0.22 | 0% |  |
| Resistance exercise | 51 | –0.83 [–1.81, 0.14] | 0.09 | 80% |  |
| Cycling | 133 | –0.64 [–1.19, –0.08] | 0.02 | 79% |  |
| **Bout Duration** |  |  |  |  | **0.62** |
| Short duration (≤3 min) | 67 | –0.40 [–1.15, 0.35] | 0.29 | 75% |  |
| Long duration (>3 min) | 133 | –0.64 [–1.19, –0.08] | 0.02 | 79% |  |
| **Total Daily Dose** |  |  |  |  | **0.0006** |
| Low dose (≤30 min/day) | 42 | –0.27 [–0.70, 0.16] | 0.22 | 0% |  |
| Moderate–low dose (31–60 min/day) | 149 | –0.45 [–0.96, 0.06] | 0.08 | 77% |  |
| Moderate–high dose (61–120 min/day) | 9 | –3.48 [–5.07, –1.90] | <0.0001 | – |  |
