# Supplementary figures and images for: Acute effects of exercise snacks on postprandial glucose and insulin metabolism in adults with obesity: a systematic review and meta-analysis
Source: Front Nutr. 2025 Nov 20;12:1708301. doi: 10.3389/fnut.2025.1708301 (PMC12677009; doi:10.3389/fnut.2025.1708301)

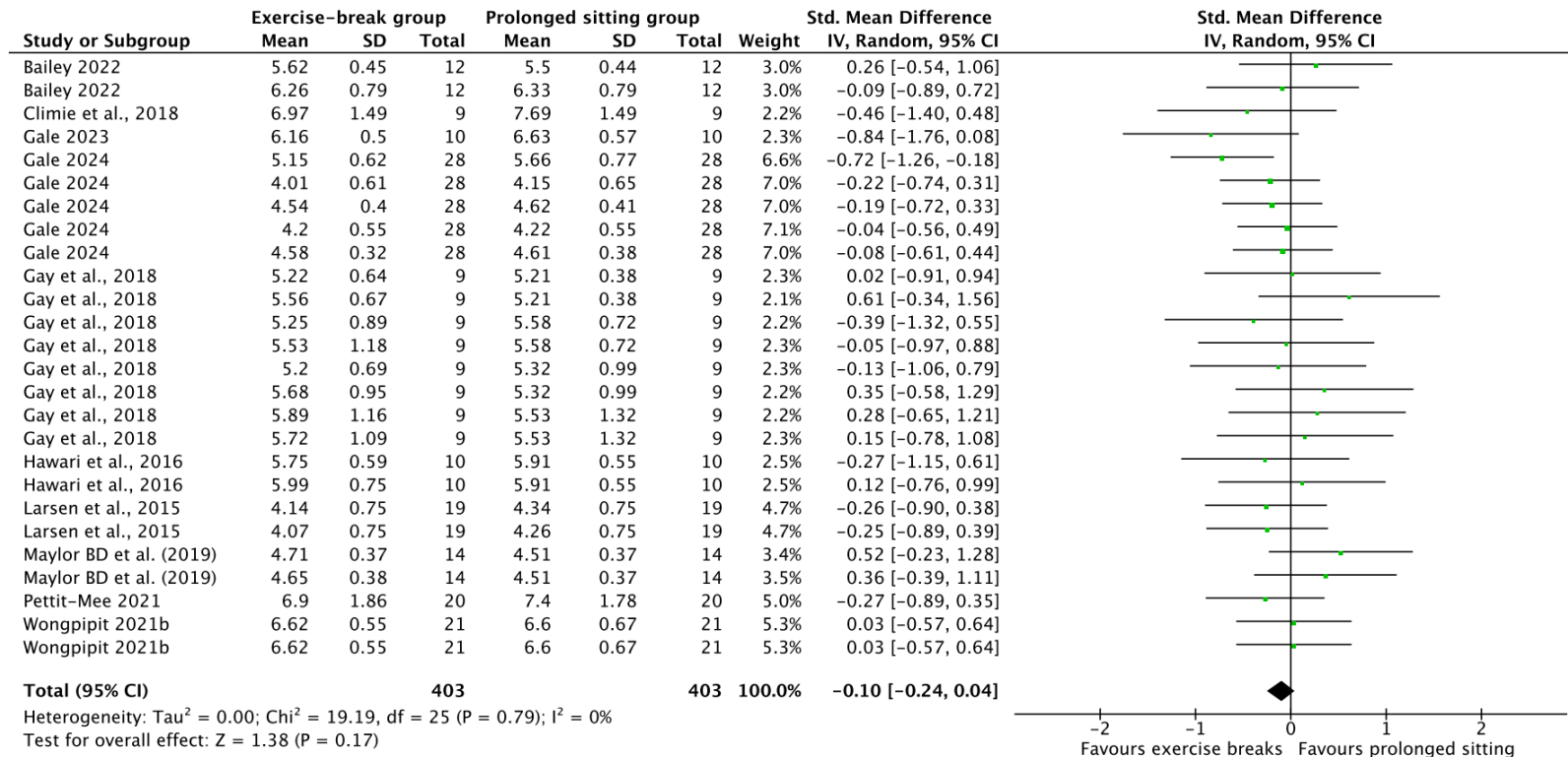

**Figures S1.** Forest plot of glucose AUC comparing exercise breaks with prolonged sitting

Supplement: Supplementary file 7 [file Image_1.pdf]
