# Supplementary material for: Acute effects of exercise snacks on postprandial glucose and insulin metabolism in adults with obesity: a systematic review and meta-analysis
Source: Front Nutr. 2025 Nov 20;12:1708301. doi: 10.3389/fnut.2025.1708301 (PMC12677009; doi:10.3389/fnut.2025.1708301)
Supplement: Supplementary file 8 [file Image_2.pdf]

	Exercise-break group			Prolonged sitting group				Std. Mean Difference
**Study or Subgroup**	**Mean**	**SD**	**Total**	**Mean**	**SD**	**Total**	**Weight**	**IV, Random, 95% CI**
Climie et al., 2018	0.6	0.1	9	1	0.1	9	4.3%	−3.81 [−5.49, −2.13]
Climie et al., 2018	0.4	0.1	9	0.7	0.1	9	5.3%	−2.86 [−4.26, −1.45]
Climie et al., 2018	1.4	0.3	9	2	0.3	9	6.3%	−1.90 [−3.07, −0.74]
Gale 2024	0.7	0.28	28	0.78	0.21	28	9.4%	−0.32 [−0.85, 0.21]
Gale 2024	0.52	0.3	28	0.38	0.22	28	9.3%	0.52 [−0.01, 1.06]
Gale 2024	0.81	0.16	28	0.69	0.18	28	9.3%	0.69 [0.15, 1.24]
Gale 2024	0.46	0.27	28	0.38	0.21	28	9.4%	0.33 [−0.20, 0.85]
Gale 2024	0.81	0.18	28	0.7	0.18	28	9.3%	0.60 [0.07, 1.14]
Gale 2024	0.83	0.38	28	0.65	0.29	28	9.3%	0.53 [−0.01, 1.06]
Gale 2024	0.89	0.2	28	0.8	0.17	28	9.4%	0.48 [−0.05, 1.01]
Gale 2024	0.71	0.38	28	0.59	0.28	28	9.4%	0.35 [−0.17, 0.88]
Gale 2024	0.85	0.14	28	0.77	0.12	28	9.3%	0.61 [0.07, 1.14]
**Total (95% CI)**			**279**			**279**	**100.0%**	**−0.08 [−0.53, 0.37]**

Heterogeneity: $Tau^2 = 0.49$; $Chi^2 = 67.71$, df = 11 ($P < 0.00001$); $I^2 = 84\%$
Test for overall effect: Z = 0.35 (P = 0.73)

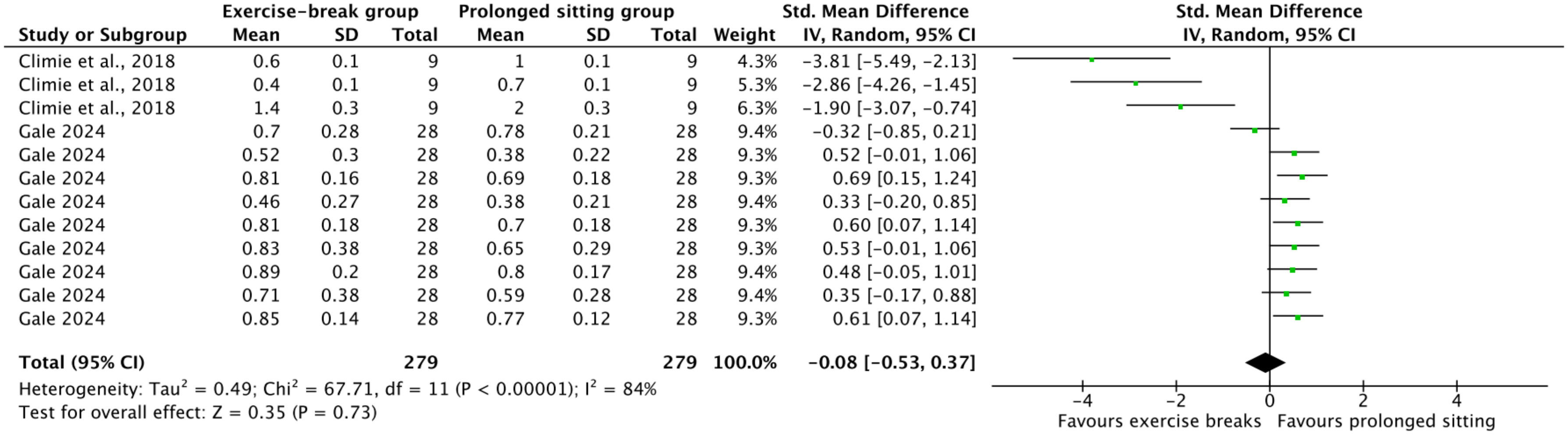

Figures S2. Forest plot of glucose variability outcomes comparing exercise breaks with prolonged sitting
