# Supplementary material for: Acute effects of exercise snacks on postprandial glucose and insulin metabolism in adults with obesity: a systematic review and meta-analysis
Source: Front Nutr. 2025 Nov 20;12:1708301. doi: 10.3389/fnut.2025.1708301 (PMC12677009; doi:10.3389/fnut.2025.1708301)
Supplement: Supplementary file 9 [file Image_3.pdf]

Study or Subgroup	Exercise-break group Mean	SD	Total	Prolonged sitting group Mean	SD	Total	Weight	Std. Mean Difference IV, Random, 95% CI
Maylor BD et al. (2019)	117.6	42	14	143.57	41.49	14	8.5%	−0.60 [−1.36, 0.16]
Larsen et al., 2015	1,309	627.681	19	1,484	710.501	19	12.0%	−0.26 [−0.89, 0.38]
Larsen et al., 2015	1,301	623.323	19	1,474	706.142	19	12.0%	−0.25 [−0.89, 0.38]
Pettit-Mee 2021	514.92	325.67	20	593.55	345.6	20	12.7%	−0.23 [−0.85, 0.39]
Hawari et al., 2016	1,974	853.81	10	2,130	891.76	10	6.4%	−0.17 [−1.05, 0.71]
Gale 2023	5,076.5	2,748.45	10	5,502.25	3,131.48	10	6.4%	−0.14 [−1.02, 0.74]
Hawari et al., 2016	2,010	1,138.42	10	2,130	891.76	10	6.4%	−0.11 [−0.99, 0.76]
Maylor BD et al. (2019)	141.68	42.25	14	143.57	41.49	14	8.9%	−0.04 [−0.78, 0.70]
Wongpipit 2021b	3,991	2,027	21	3,884	1,770	21	13.4%	0.06 [−0.55, 0.66]
Wongpipit 2021b	4,013	1,978	21	3,884	1,770	21	13.4%	0.07 [−0.54, 0.67]
**Total (95% CI)**			**158**			**158**	**100.0%**	**−0.16 [−0.38, 0.07]**

Heterogeneity: $Tau^2 = 0.00$; $Chi^2 = 2.66$, df = 9 (P = 0.98); $I^2 = 0\%$

Test for overall effect: Z = 1.38 (P = 0.17)

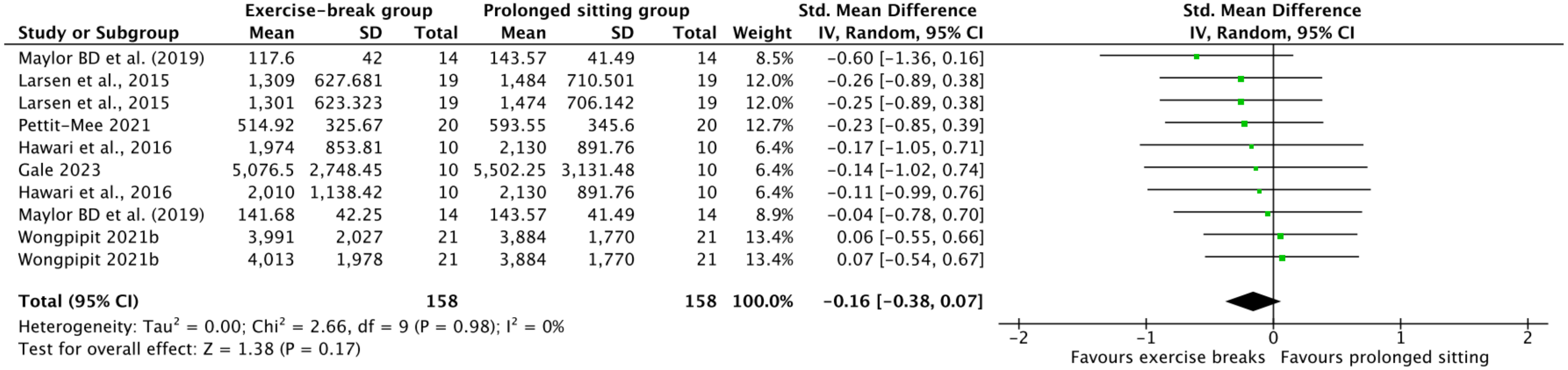

Figures S3. Forest plot of insulin AUC outcomes comparing exercise breaks with prolonged sitting
