# Supplementary material for: Acute effects of exercise snacks on postprandial glucose and insulin metabolism in adults with obesity: a systematic review and meta-analysis
Source: Front Nutr. 2025 Nov 20;12:1708301. doi: 10.3389/fnut.2025.1708301 (PMC12677009; doi:10.3389/fnut.2025.1708301)
Supplement: Supplementary file 10 [file Image_4.pdf]

Study or Subgroup	Exercise-break group Mean	SD	Total	Prolonged sitting group Mean	SD	Total	Weight	Std. Mean Difference IV, Random, 95% CI
Climie et al., 2018	1.3	0.19	9	1.7	0.19	9	1.3%	−2.01 [−3.19, −0.82]
Gale 2024	−0.18	3.17	28	0.08	1.3	28	6.0%	−0.11 [−0.63, 0.42]
Gale 2024	0.62	3.14	28	1.37	1.18	28	5.9%	−0.31 [−0.84, 0.22]
Gale 2024	−0.39	3.14	28	0.02	1.22	28	6.0%	−0.17 [−0.69, 0.36]
Gale 2024	0.01	3.31	28	0.39	1.38	28	6.0%	−0.15 [−0.67, 0.38]
Gale 2024	0.02	3.37	28	0.29	1.34	28	6.0%	−0.10 [−0.63, 0.42]
Hawari et al., 2019	0.87	0.51	14	0.92	0.64	14	3.2%	−0.08 [−0.83, 0.66]
Hawari et al., 2019	1.11	0.54	14	1.28	0.82	14	3.2%	−0.24 [−0.98, 0.51]
Hawari et al., 2019	0.9	0.41	14	0.99	0.64	14	3.2%	−0.16 [−0.90, 0.58]
Hoffmann 2024	0.27	0.26	17	0.4	0.25	17	3.8%	−0.50 [−1.18, 0.19]
Hoffmann 2024	0.35	0.26	17	0.41	0.23	17	3.8%	−0.24 [−0.91, 0.44]
Hoffmann 2024	0.59	0.26	17	0.73	0.32	17	3.8%	−0.47 [−1.15, 0.21]
Larsen et al., 2015	1.9	11.02	19	3	11.81	19	4.3%	−0.09 [−0.73, 0.54]
Larsen et al., 2015	2.2	11.02	19	2.9	12.76	19	4.3%	−0.06 [−0.69, 0.58]
Rodriguez-Hernandez et al. (2018)	1.59	3.55	10	2.15	4.05	10	2.4%	−0.14 [−1.02, 0.74]
Rodriguez-Hernandez et al. (2018)	1.59	2.29	10	2.15	4.05	10	2.4%	−0.16 [−1.04, 0.72]
Smith et al., 2021	1.3	1.51	8	−0.6	2	8	1.7%	1.01 [−0.05, 2.07]
Smith et al., 2021	−0.3	0.54	8	−0.1	0.32	8	1.9%	−0.43 [−1.42, 0.57]
Wanders et al., 2021	−0.2	0.52	18	−0.09	0.53	18	4.1%	−0.20 [−0.86, 0.45]
Wanders et al., 2021	0.47	1.3	18	0.43	1.41	18	4.1%	0.03 [−0.62, 0.68]
Wanders et al., 2021	0.44	0.87	16	0.2	0.99	18	3.8%	0.25 [−0.43, 0.93]
Wanders et al., 2021	−0.25	0.49	19	−0.48	0.67	17	4.0%	0.39 [−0.27, 1.05]
Wanders et al., 2021	0.98	1.43	17	1.13	1.55	18	4.0%	−0.10 [−0.76, 0.57]
Wanders et al., 2021	−0.68	0.4	18	−0.57	0.43	16	3.8%	−0.26 [−0.94, 0.42]
Wanders et al., 2021	0.05	0.61	16	−0.39	0.52	17	3.5%	0.76 [0.05, 1.47]
Wanders et al., 2021	−0.62	0.44	16	−0.72	0.73	16	3.7%	0.16 [−0.53, 0.86]
**Total (95% CI)**			**454**			**454**	**100.0%**	**−0.11 [−0.25, 0.03]**

Heterogeneity: $Tau^2 = 0.01$; $Chi^2 = 27.83$, df = 25 (P = 0.32); $I^2 = 10\%$

Test for overall effect: Z = 1.49 (P = 0.14)

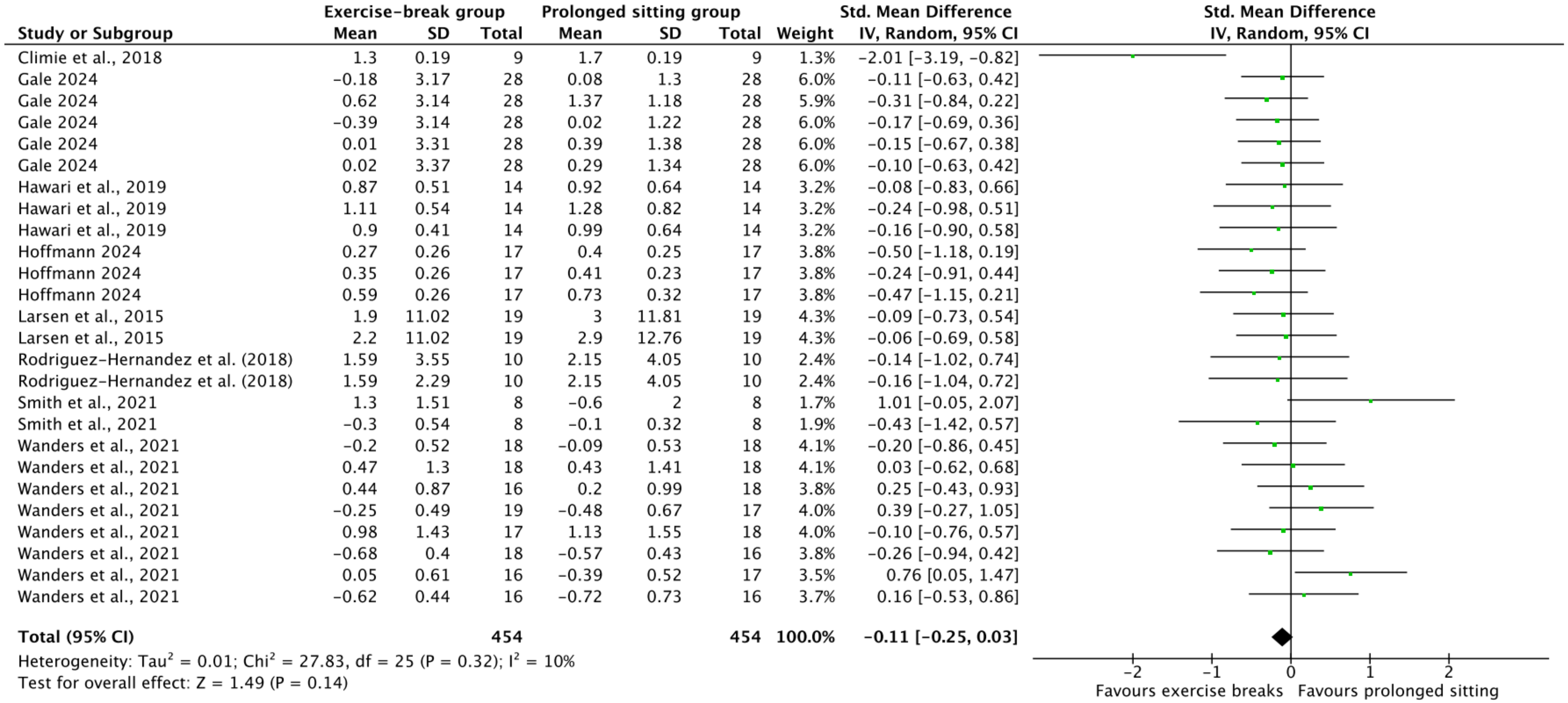

Figures S4. Forest plot of mean glucose outcomes comparing exercise breaks with prolonged sitting
