# Supplementary material for: Acute effects of exercise snacks on postprandial glucose and insulin metabolism in adults with obesity: a systematic review and meta-analysis
Source: Front Nutr. 2025 Nov 20;12:1708301. doi: 10.3389/fnut.2025.1708301 (PMC12677009; doi:10.3389/fnut.2025.1708301)
Supplement: Supplementary file 11 [file Image_5.pdf]

Study or Subgroup	Exercise-break group Mean	SD	Total	Prolonged sitting group Mean	SD	Total	Weight	Std. Mean Difference IV, Random, 95% CI
Climie et al., 2018	48.3	8.71	9	80.17	8.71	9	4.2%	−3.48 [−5.07, −1.90]
Hawari et al., 2019	62.9	36.73	14	79.7	51.77	14	7.5%	−0.36 [−1.11, 0.38]
Hawari et al., 2019	67.2	37.1	14	75.5	51.39	14	7.5%	−0.18 [−0.92, 0.56]
Hawari et al., 2019	62.6	33.75	14	74.1	48.41	14	7.5%	−0.27 [−1.01, 0.48]
Smith et al., 2021	1.3	4.32	8	0.3	4.81	8	6.4%	0.21 [−0.78, 1.19]
Smith et al., 2021	20.2	44.11	8	−5.8	24.66	8	6.3%	0.69 [−0.33, 1.71]
Wanders et al., 2021	31.25	13.24	17	31.66	15.91	17	7.8%	−0.03 [−0.70, 0.64]
Wanders et al., 2021	30.1	6.8	16	38.41	12.08	15	7.5%	−0.83 [−1.57, −0.09]
Wanders et al., 2021	−0.39	2.37	17	8.51	8	17	7.4%	−1.47 [−2.24, −0.70]
Wanders et al., 2021	2.79	2.02	16	7.15	1.69	17	6.8%	−2.29 [−3.19, −1.39]
Wanders et al., 2021	−2.37	1.45	17	−1.05	1.8	15	7.6%	−0.79 [−1.52, −0.07]
Wanders et al., 2021	−2.58	1.77	16	−2.07	1.49	17	7.8%	−0.30 [−0.99, 0.38]
Wanders et al., 2021	58.27	22.97	18	53.15	16.74	17	7.9%	0.25 [−0.42, 0.91]
Wanders et al., 2021	64.98	18.74	16	63.16	16.94	18	7.8%	0.10 [−0.57, 0.77]
**Total (95% CI)**			**200**			**200**	**100.0%**	**−0.54 [−0.97, −0.10]**

Heterogeneity: $Tau^2 = 0.51$; $Chi^2 = 54.56$, df = 13 ($P < 0.00001$); $I^2 = 76\%$

Test for overall effect: Z = 2.43 (P = 0.02)

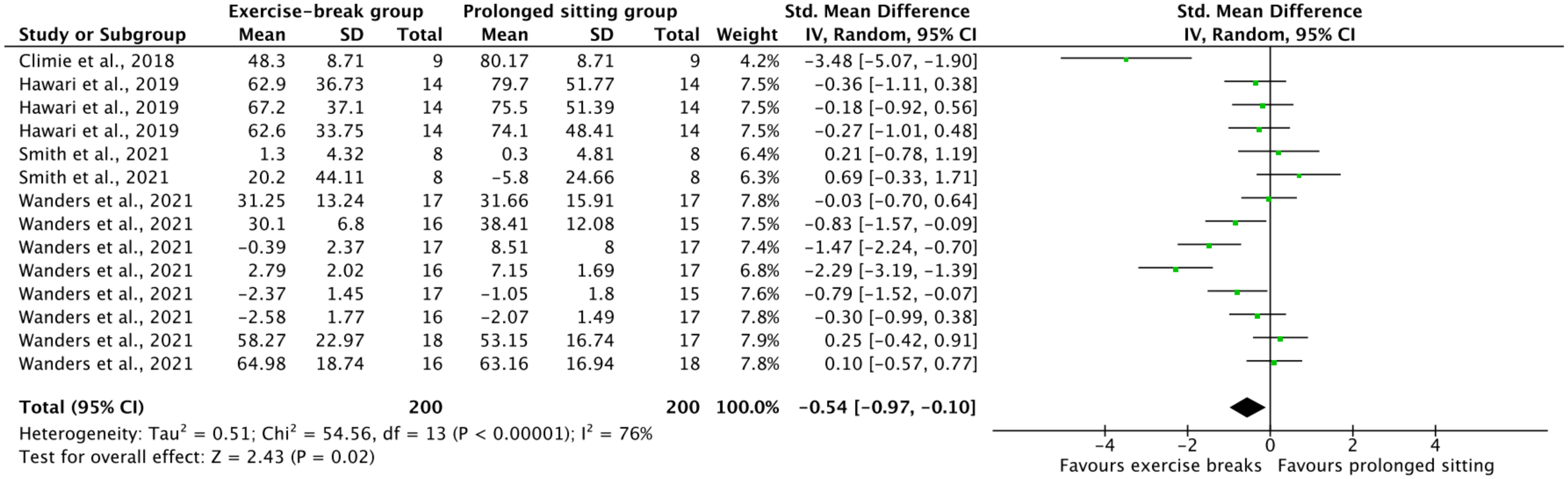

Figures S5. Forest plot of mean insulin outcomes comparing exercise breaks with prolonged sitting
